# Ultrasound-assisted bromination of indazoles at the C3 position with dibromohydantoin†

**DOI:** 10.1039/d2ra06867b

**Published:** 2022-12-22

**Authors:** Shengneng Ying, Xingru Liu, Tao Guo, Xuan Li, Min Zhou, Xia Wang, Mengxue Zhu, Hongmei Jiang, Qing-Wen Gui

**Affiliations:** College of Chemistry and Materials Science, Hunan Agricultural University Changsha 410082 Hunan P. R. China gqw1216@hunau.edu.cn wangxia@hunau.edu.cn

## Abstract

Bromoaryl compounds have attracted great attention in organic chemistry, especially for the synthesis of pharmaceutical intermediates. Herein, we demonstrated a novel and efficient bromination protocol of indazoles *via* C–H bond cleavage to give site-specific 3-bromide products that could be further employed as synthetic blocks to prepare drugs. The reaction used DBDMH as a bromine source, tolerated a wide range of indazoles, and finished in 30 min under mild, ultrasound-assisted conditions. Besides, preliminary mechanistic studies revealed that this approach was not a radical process.

## Abstract 2

Bromoaryl compounds have attracted great attention in organic chemistry, especially for the synthesis of pharmaceutical intermediates.

## Graphical abstract

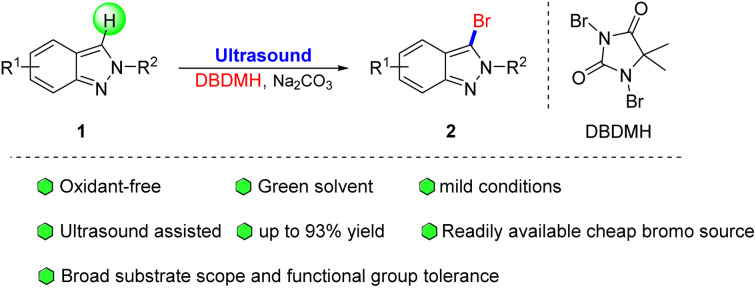

## Introduction

**Fig. 1:**
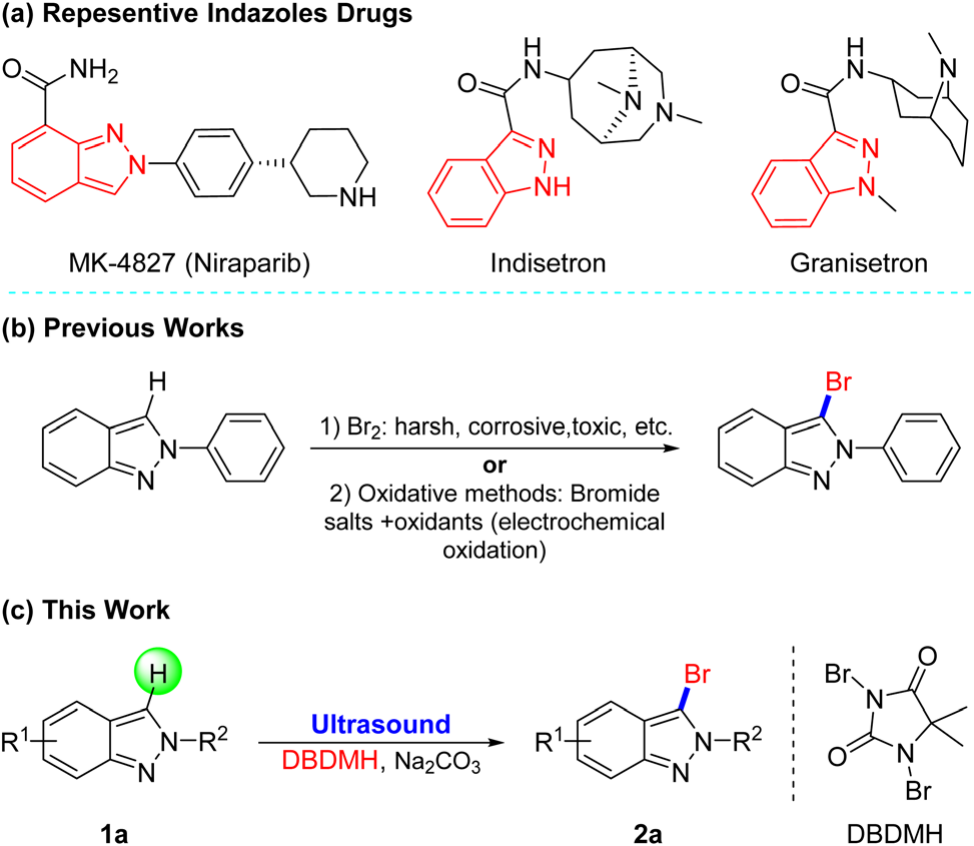
(a) Representative drugs containing indazoles. Previous works (b) and this work (c) for bromination of indazole derivatives.

Heterocyclics frequently constitute the core moiety of various pharmaceuticals.^1^ Among them, indazoles, possessing a wide range of biological activities such as antitumor, antimicrobial, and anti-inflammatory, are a critical class of *N*-heterocyclic molecules.^2^ For example, MK-4827 (ref. 3) as a PARP1 and PARP2 inhibitor has a 1*H*-indazole skeleton, and Indisetron^4^ and Granisetron,^5^ as antiemetics that can be used to treat nausea and emesis following chemotherapy, have an indazole construction (Fig. 1a). However, the limited methods for the functionalization of indazoles cannot meet the demands for structural diversity.

Due to the general application of halogenated indazoles as building blocks of the synthesis of drugs, dyes, and functional materials,^6^ it's significant to develop novel and efficient halogenation approaches, especially bromination which is flexible for post-group-transformation,^7^ Conventionally, 3-halogenated-2*H*-indazoles are prepared from 2*H*-indazoles with Br_2_ in acetic acid. However, these halogenated approaches often need high temperature (*ca* 120 °C) for activating reaction, plus Br_2_ is toxic to humans and difficult to operate owing to its high volatility, and byproducts are unavoidable (Fig. 1b).^8^ To avoid the use of the liquid bromine, a variety of oxidative protocols have then been disclosed through *in situ* oxidation of Br^−^ to Br^+^.^9^ For instance, the group of Li and Shen disclosed the electrochemical oxidative halogenation of 2*H*-indazoles under mild conditions, recently.^9*a*^ Despite the fruitful stoichiometric methods, the more attractive and novel halogenation technology is still in high demand.

Ultrasonic waves have gradually developed as a potent tool in organic synthesis, owing to their merits such as short reaction time, mild reaction conditions, good selectivity, and highly efficient.^10^ It can accelerate the thermal motion of molecules, further speeding up the mass and heat transfer between chemicals. Dating back to 1950, Renaud first published a paper about using ultrasound to prepare organometallic reagents.^11^ In 1998, Luche and Bianchi concretely described the application and potential of ultrasound to organic synthesis in “Synthetic Organic Sonochemistry”,^12^ and then ultrasound was gradually accepted and developed. Presently, ultrasound is a helpful technique for activating and accelerating chemical processes.^13^

1,3-Dibromo-5,5-dimethyl hydantoin (DBDMH) belongs to the group of cost-effective *N*-halamine disinfectants, which is becoming increasingly popular due to its long-term stability in dry storage or in a wide pH range of aqueous solutions, it's safety for humans and the environment, and their ability to rapidly kill microorganisms.^14^ Meanwhile, relative to other bromine sources, DBDMH is less corrosive, more stable and cheaper.^15^ Furthermore, DBDMH is among the more established, commercially available bromine carry that is gradually attracting attention for being safe, stable, easily-handled solids that can be utilized under mild conditions for highly selective organic transformations.^16^ Thus, in the context of our group's continued interest in improving and developing environmentally acceptable synthetic methods,^17^ we now describe the first practical and simple design for selective 3-bromination of 2*H*-indazoles with DBDMH in a green solvent accelerated by ultrasonic irradiation, which produced 3-brominated products in very high yields within a short time (Fig. 1c).

## Results and discussion

Inspired by seminal works in bromination we started our investigation by designing bromination of 1*H*-indazole with an appropriate brominated reagent that would represent an efficient and mild strategy that could overcome many limitations of current methods. As shown in Table 1, we first screened different solvents such as DMF, MeCN, THF, DCM, EA, and EtOH with Na_2_CO_3_ as base and to afford 2a at 80 °C for 12 h (entry 1–6), while EtOH was considered to be the competent solvent among them. To decrease reaction time and temperature, ultrasound was introduced to replace conditional stir,^18^ excitedly, which greatly increased yields and transformation efficiency (entry 7–9). Bases were also included in the scope of optimization, like Et_3_N (entry 10), NaOAc (entry 11) and K_2_CO_3_(entry 12), all of which can promote the reaction process, and K_2_CO_3_ obtained similar results to Na_2_CO_3_. Therefore, the optimized experimental conditions were determined as follows: Na_2_CO_3_ as base, EtOH as solvent, and ultrasound for 0.5 h at 40 °C. The ultrasound-assisted reaction was screened with various bromine sources, such as NBS, NaBr and HBr (entries 13–15). There bromine sources did not provide any satisfactory result; rather, a very sluggish reaction rate or no reaction was observed in each case.

**Table 1:** Optimization of reaction conditions^a^

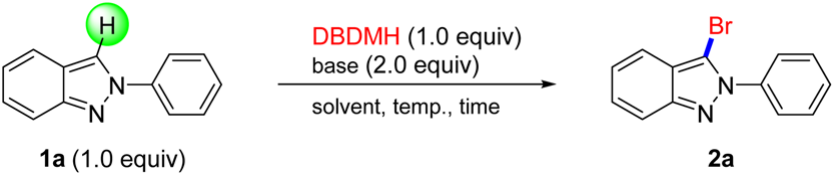					
Entry	Base	Solvent	Temp.(°C)	Time (h)	Yield^b^ (%)
1	Na_2_CO_3_	DMF	80	12	76
2	Na_2_CO_3_	CH_3_CN	80	12	72
3	Na_2_CO_3_	THF	80	12	88
4	Na_2_CO_3_	DCM	80	12	68
5	Na_2_CO_3_	EtOAc	80	12	80
6	Na_2_CO_3_	Toluene	40	12	75
7^c^	Na_2_CO_3_	EtoH	40	0.5	92
8^c^	Na_2_CO_3_	EtoH	40	0.15	88
9^c^	Na_2_CO_3_	EtoH	30	0.5	87
10^c^	Et_3_N	EtoH	40	0.5	23
11^c^	NaOAc	EtoH	40	0.5	29
12^c^	K_2_CO_3_	EtoH	40	0.5	91
13^d^	Na_2_CO_3_	EtoH	30	0.5	Trace
14^e^	Na_2_CO_3_	EtoH	30	0.5	35
15^f^	Na_2_CO_3_	EtoH	30	0.5	NR

a Reaction conditions: 1a (0.2 mmol), DBDMH (0.2 mmol), base (0.4 mmol).

b Isolated yield.

c Ultrasound instead of stirring.

d NaBr instead of DBDMH.

e NBSr instead of DBDMH.

f HBr instead of DBDMH.

**Scheme 1:**
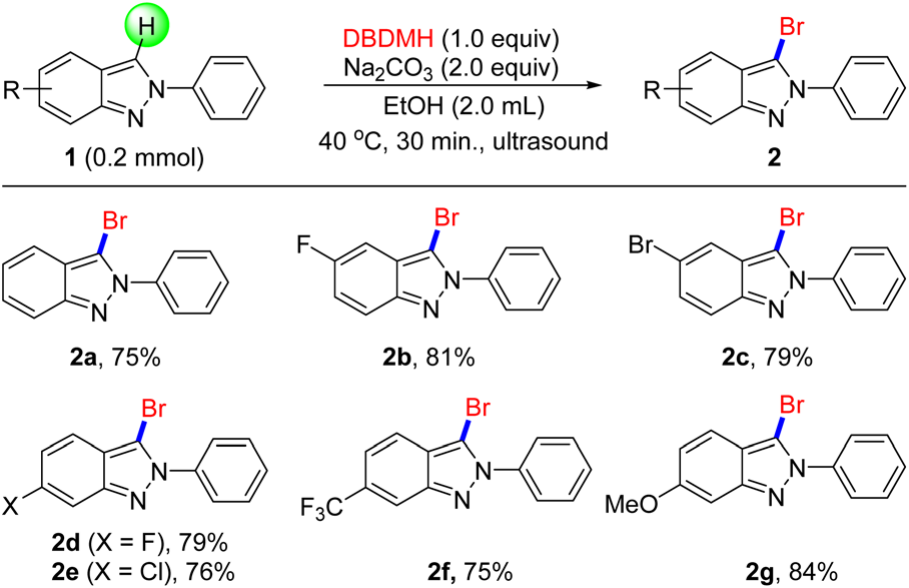
The Bromination of 2*H*-indazoles. ^*a*^Reaction conditions: 1 (0.2 mmol), DBDMH (0.2 mmol), Na_2_CO_3_ (0.4 mmol), 40 °C, EtOH (2.0 mL), ultrasonic (40 kHz/50 W) 30 min.

**Scheme 2:**
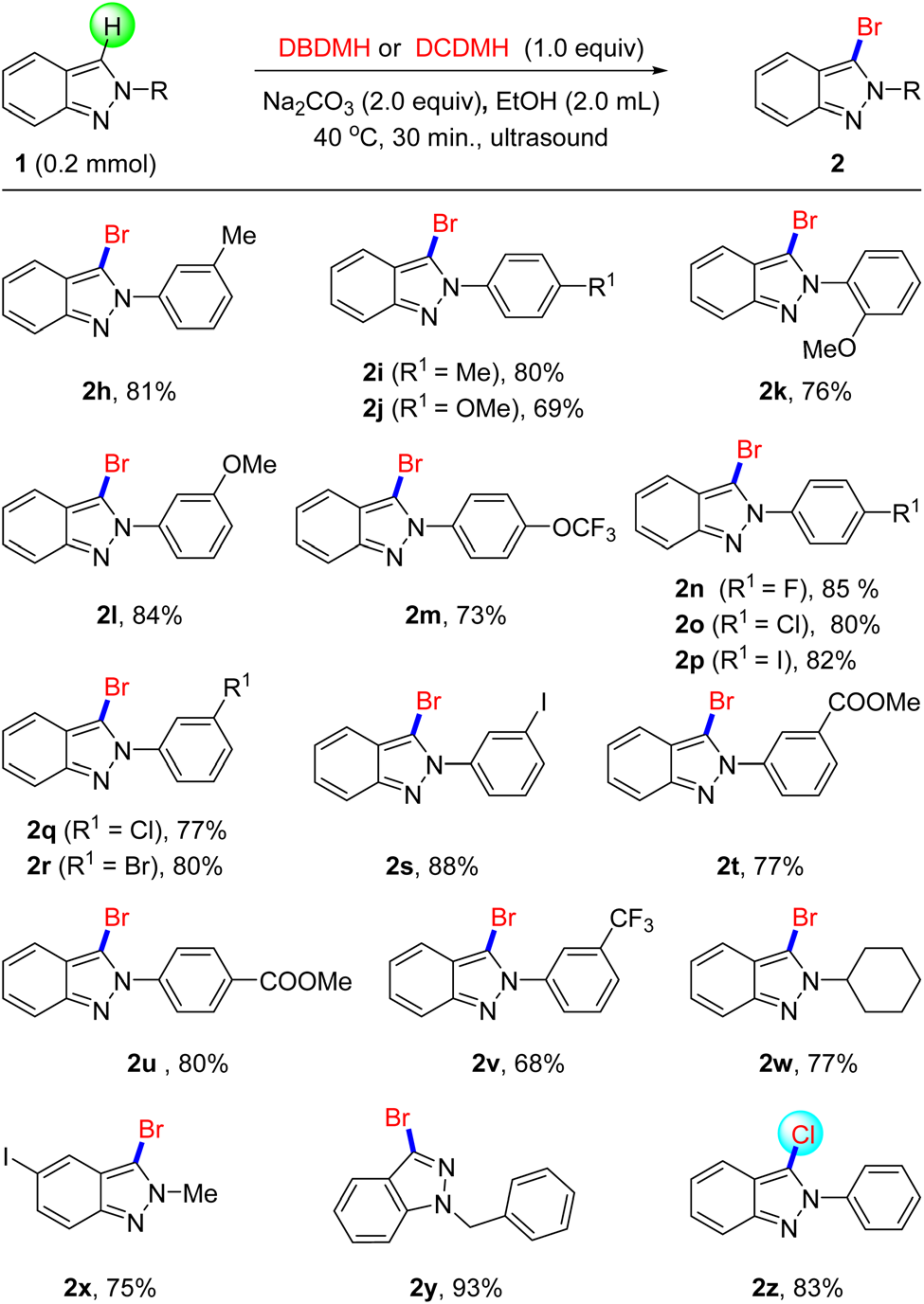
The Halogenation of Indazoles. ^*a*^Reaction conditions: 1 (0.2 mmol), DBDMH (0.2 mmol), Na_2_CO_3_ (0.4 mmol), 40 °C, EtOH (2.0 mL), ultrasonic 30 min ^*b*^DCDMH (0.2 mmol).

With the optimal conditions in hand, the generality of our newly developed bromination protocol was next investigated. Firstly, a series of electron-deficient 2*H*-indazoles were examined, including halides such as fluorine (Scheme 1, 2b, 2d), bromine (2c) and chlorine (2e), all of which proceeded smoothly to obtain target products in mild to favorable yield under standard conditions. For electron-rich groups, 2f was prepared with 81% yield that was not affected by –OMe. Notably, the bromination of 2g afforded brominated 2*H*-indazoles, even which included a strong electron-withdrawing group CF_3_.

Encouraged by the exciting results, we then evaluated the activities of 2-substituted indazole derivatives that reacted with DBDMH in standard conditions. As shown in Scheme 2, the bromination, of 2*H*-indazoles substituted by electron-donating groups such as –Me and –OMe, succeeded to afford the 3-brominated 2h–2l in mild to favorable yield, which also reported that the substitution position of groups had less effect on bromination reactivity. Subsequently, the electron-withdrawing group OCF_3_ was found to proceed smoothly in this transformation (2l), and acquired 73% yield. Then, we estimated the bromination of halogen-substituted 2*H*-indazoles derivatives. Under the standard conditions, fluorine, chlorine, bromine, and iodine replaced the hydrogen of the benzene, which brominated successfully to obtain the corresponding products with favorable yields (2n–2s). It is worth noting that the bromination of ester group substituted derivatives performed favorably to access brominated products 2t–2u with good yields and excellent selectivity. With regard to strong electron withdrawing group CF_3_, 2v was prepared with a slightly lower yield compared without substituents. Surprisingly, when we used cyclohexyl substituted benzene coupled with 2*H*-indazole, a good bromination effect was achieved (2w). Gratifyingly, methylated 1*H*-indazole was also carried out smoothly to generate an objective product (2x), and the transformation was unaffected by halogen iodine. Moreover, 1*H*-indazole substituted by benzyl also presented excellent activity to acquire 2y (93%). In addition, we tried to employ DCDMH to replace DBDMH in this reaction, as expected, chlorination could proceed smoothly under the traditional conditions (2z). All of these experiment results illustrated that this method possessed broad applicability and group tolerance.

**Scheme 3:**
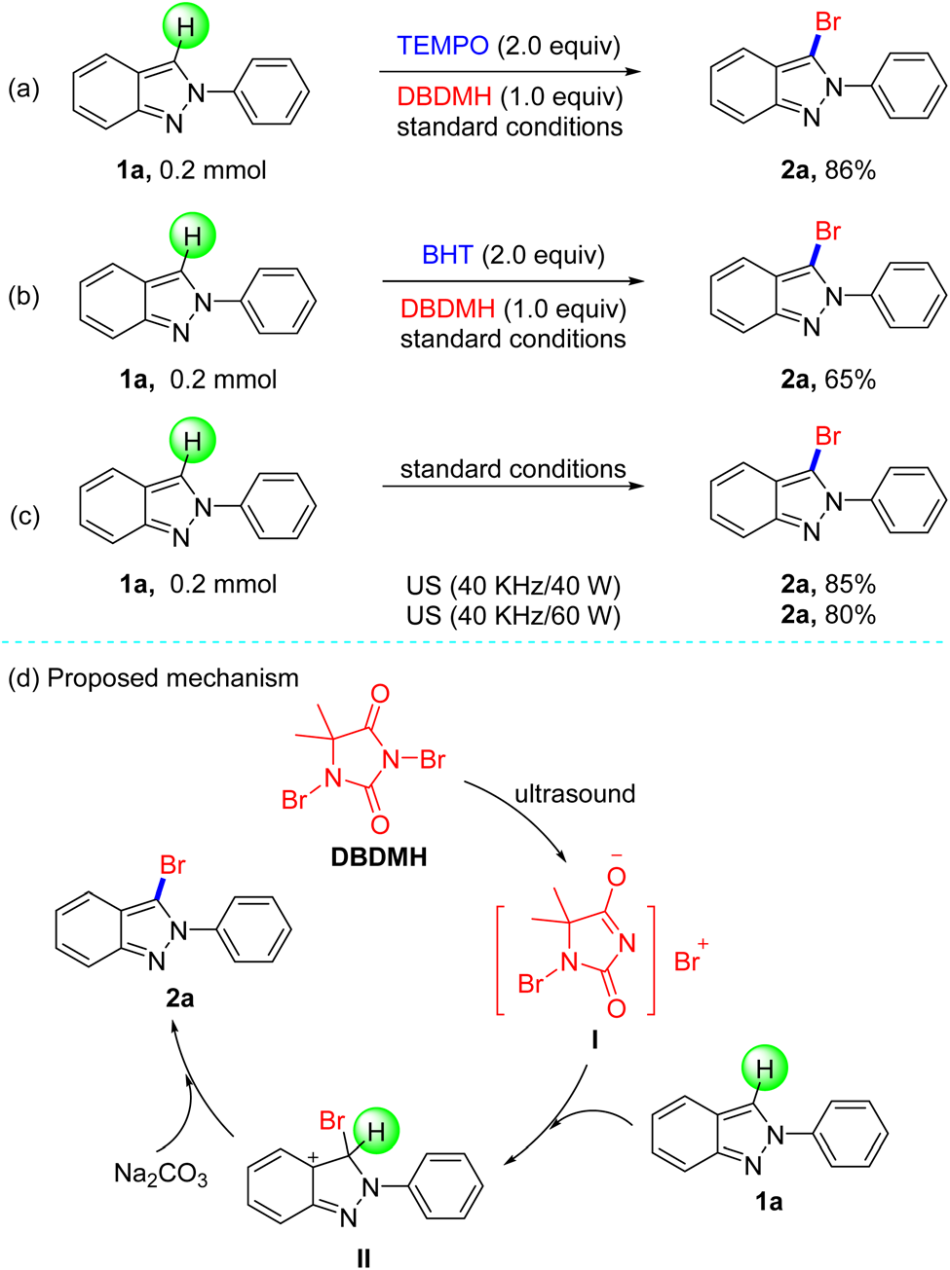
Control Experiments and Mechanism study.

Based on the experiment results, we next investigated the mechanistic information of this transformation with some control experiments in Scheme 3. These results depicted that 2-phenyl-2*H*-indazole (1a) could be brominated in the presence of radical scavengers such as 2,2,6,6-tetramethylpiperidine-1-oxyl (TEMPO) and 2,6-di-*tert*-butyl-4-methyl phenol (BHT), and gave 86% and 65% yield, respectively (Scheme 3a and b). In order to show the cavitation effect of ultrasonic irradiation, the employment of other energies (40 kHz/40 W or 40 kHz/60 W) produced 2a in slightly lower yields (Scheme 3c). In view of the primary mechanism data and previous reports, we speculate this method is not a radical process, and the proposed reaction pathway is presented in Scheme 3d. Firstly, the cleaving of DBDMH by ultrasonic irradiation generated a bromo ion and I,^19^ which added to 1a to form intermediate II. Finally, product 2a was obtained from II, followed by the abstraction of a hydrogen atom by base.

**Scheme 4:**
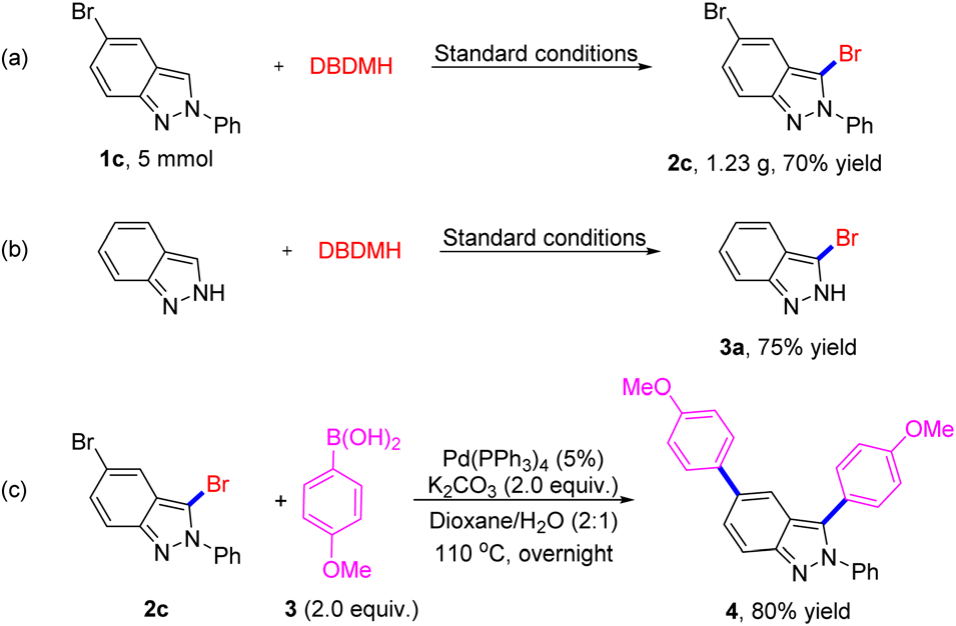
Further Application.

To demonstrate the practicality of this approach, we conducted the gram-scale iodination of 2*H*-indazole under the optimized conditions, which gave the desired product 3-bromo-2*H*-indazole (2c) in a 70% yield (Scheme 4a). Considering the good selectivity of this method and the importance of 2*H*-indazoles in pharmaceuticals, we take advantage of products for further transformation to extend the application scope. It is noteworthy to mention that indazole can provide side products due to competitive reactive sites, but generated product 3a without having any impact on yield (75%). As reported that Br is an excellent group for coupling reaction, we chose 2c and aryl boronic acid 3 as substrates for cross-coupling reaction, expectedly, which was conducted successfully under Pd catalysis (Scheme 4c, 4, 80% isolated yield). The application extension results suggest the broad applicability potentiality of our method.

## Conclusions

In conclusion, we have successfully developed an ultrasound-assisted, efficient, and rapid bromination approach *via* DBDMH as a bromine source for the synthesis of 3-Br-indazoles. This reaction underwent an ultrasound-assisted C–H bond cleavage and C–Br bond formation process, representing one of the few C–Br bond construction reactions under ultrasound waves. Furthermore, a wide range of functionalized indazoles is compatible with mild reaction conditions. The mechanism investigation revealed that the reaction is not a complete radical process. More importantly, the brominated products can be applied for further application, providing a potential strategy for the synthesis of pharmaceutical intermediates. Further investigation of related halogenation reactions is carried on studying in our group.

## Conflicts of interest

There are no conflicts to declare.

## Supplementary Material

RA-013-D2RA06867B-s001
