# Supplementary material for: Ultrasound-assisted bromination of indazoles at the C3 position with dibromohydantoin
Source: RSC Adv. 2022 Dec 22;13(1):581–5. doi: 10.1039/d2ra06867b (PMC9773018; doi:10.1039/d2ra06867b)

Electronic Supplementary Material (ESI) for RSC Advances.
This journal is © The Royal Society of Chemistry 2022

Supplementary information for

Ultrasound-assisted Bromination of Indazoles at C3 Position with Dibromohydantoin

Shengneng Ying,[#] Xingru Liu,[#] Tao Guo, Xuan Li, Min Zhou, Xia Wang,* Mengxue Zhu, Hongmei Jiang, Qing-wen Gui*

Table of Content:

1. **General Information..S2**

2. **Experimental Section……..................…..S2**

3. **Characterization data of products...S3**

4. **^{1}H and ^{13}C NMR spectra of products……………….……………….……S10**

1. General Information

All reactions were carried out in anhydrous solvent and commercially available reagents were used as received unless otherwise stated. Analytical thin layer chromatography (TLC) was performed on precoated aluminium-backed silica gel 60 F_{254} plates (EMD Millipore, 200 μm thickness). TLC plates were visualized with ultraviolet light and treatment with $KMnO_4$ or vanillin stains followed by heating. Flash column chromatography was performed using Tsingtao silica gel (200-300). ^{1}H and ^{13}C NMR spectras were recorded on a Bruker Avance DRX-400 spectrometers; chemical shifts (δ) are given in ppm and calibrated using the signal of residual undeuterated solvent as internal reference ($CHCl_3$: δ_H =7.26 ppm and δ_C = 77.16 ppm). Data for ^{1}H NMR and ^{13}C NMR are reported as follows: chemical shift (δ, ppm), multiplicity, integration, and coupling constant (Hz).

2. Experimental Section

General experimental procedures for 2a

To an equipped with a magnetic stir bar oven-dried reaction tube (10 mL) was charged with 2-phenyl-2H-indazole (**1a**) (1.0 equiv, 0.2 mmol, 38.80 mg), 1,3-dibromo-5,5-dimethylimidazolidine-2,4-dione (DBDMH) (1.0 equiv, 0.2 mmol, 56.60 mg), Na_2CO_3 (2.0 equiv, 0.4 mmol, 42.40 mg) and EtOH (2.0 mL). The mixture was reacted in the ultrasonic instrument for 30 mins at 40 °C. Then the mixture was diluted with AcOEt, filtered by a short silica gel column, and washed with AcOEt. The crude mixture was concentrated in vacuo, and then purified by flash column chromatography on silica gel to afford **2a** in 75% yield (40.80 mg).

3. Characterization data of products

3-bromo-2-phenyl-2H-indazole (2a)[1]

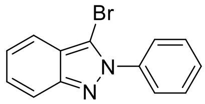

2a was obtained in 75% (40.80 mg) as a yellow solid;

^{1}H NMR (400 MHz, $CDCl_3$): *δ* 7.75 (d, *J* = 8.8 Hz, 1H), 7.69-7.67 (m, 2H), 7.60-7.51 (m, 4H), 7.39-7.35 (m, 1H), 7.20-7.16 (m, 1H); ^{13}C NMR (100 MHz, $CDCl_3$) ppm: δ 149.26, 139.30, 129.33, 129.15, 127.70, 126.25, 123.07, 122.97, 119.79, 118.25, 106.27 ppm. MS:m/z 272 (M+).

3-bromo-5-fluoro-2-phenyl-2H-indazole (2b)[1]

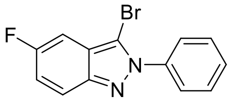

2b was obtained in 81% (46.98 mg) as a white solid;

^{1}H NMR (400 MHz, $CDCl_3$) δ 7.74 (q, *J* = 7.2 Hz, 1H), 7.68 (d, *J* = 6.8 Hz, 2H), 7.58-7.53 (m, 3H), 7.20-7.14 (m, 2H) ppm; ^{13}C NMR (101 MHz, $CDCl_3$): δ 160.17, 157.76, 146.57, 139.15, 129.29 (d, *J* = 26.0 Hz), 126.08, 122.40 (d, *J* = 12.0 Hz), 122.55 (d, *J* = 12.0 Hz), 119.30 (d, *J* = 29.0 Hz), 105.92 (d, *J* = 9.0 Hz), 102.30 (d, *J* = 25.0 Hz) ppm. MS:m/z 290 (M+).

3,5-dibromo-2-phenyl-2H-indazole (2c)[1]

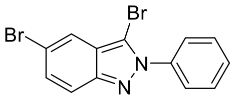

2c was obtained in 79% (55.29 mg) as a yellow solid;

^{1}H NMR (400 MHz, $CDCl_3$): δ 7.76 (s, 1H), 7.65 (t, *J* = 6.8 Hz, 3H), 7.57-7.52 (m, 3H), 7.40 (dd, *J* = 9.2 Hz, 1.6 Hz, 1H) ppm; ^{13}C NMR (101 MHz, CDCl3): δ 139.04, 131.47, 129.61, 129.27, 126.16, 124.17, 122.00, 120.08, 116.56, 105.64 ppm. MS:m/z 350 (M+).

3-bromo-6-fluoro-2-phenyl-2H-indazole (2d)

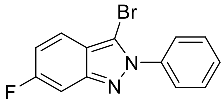

2d was obtained in 79% (45.82 mg) as a yellow solid;

^{1}H NMR (400 MHz, $CDCl_3$): δ 7.65 (d, *J* = 7.2 Hz, 2H), 7.58-7.51 (m, 4H), 7.32 (dd, *J* = 9.6 Hz, 1.6 Hz, 1 H), 6.98 (td, *J* = 9.2 Hz, 2.0 Hz, 1H) ppm; ^{13}C NMR (101 MHz, $CDCl_3$): δ 163.81, 161.37, 149.00 (d, *J* = 13.0 Hz) 139.15, 129.30 (d, *J* = 26.0 Hz), 126.14, 121.90 (d, *J* = 10.7 Hz), 120.33,

115.00 (d, J = 28.7 Hz), 107.02, 101.33 (d, J = 23.9 Hz) ppm. HRMS (ESI) m/z calcd. for $C_{13}H_8BrFN_2$ [M+H]+: 290.9928, found 290.9921.

***3-bromo-6-chloro-2-phenyl-2H-indazole (2e)*[1]**

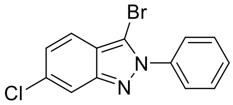

2e was obtained in 76% (46.51 mg) as a yellow solid

^{1}H NMR (400 MHz, $CDCl_3$): δ 7.73 (s, 1H), 7.65 (d, J = 6.8 Hz, 2H), 7.56-7.50 (m, 4H), 7.12 (dd, J = 9.2 Hz, 1.6 Hz, 1H) ppm; ^{13}C NMR (101 MHz, $CDCl_3$): δ 149.25, 139.05, 133.68, 129.58, 129.26, 126.16, 124.67, 121.49, 121.18, 117.16, 107.02 ppm. MS:m/z 307 (M+).

3-bromo-2-phenyl-6-(trifluoromethyl)-2H-indazole (2f)

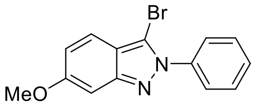

2f was obtained in 75% (51.00 mg) as a white solid;

^{1}H NMR (400 MHz, $CDCl_3$): δ 8.08 (s, 1H), 7.73-7.66 (m, 3H), 7.61-7.56 (m, 3H), 7.27 (dd, J = 8.8 Hz, 1.2 Hz, 1H) ppm; ^{13}C NMR (101 MHz, $CDCl_3$): δ 147.79, 139.03, 129.87, 129.37, 126.22, 124.01, 121.42, 118.92 (q, J = 8.7 Hz), 116.97 (q, J = 14.8 Hz), 107.29, 31.57 ppm. HRMS (ESI) m/z calcd. for $C_{14}H_{12}$BrN2O [M+H]+: 303.0128, found 303.0130.

3-bromo-6-methoxy-2-phenyl-2H-indazole (2g)

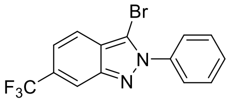

2g was obtained in 84% (50.74 mg) as a brown oil;

^{1}H NMR (400 MHz, $CDCl_3$): δ 7.66 (dd, J = 8.4 Hz, 1.6 Hz, 2H), 7.56-7.50 (m, 5H), 7.05 (d, J = 9.2 Hz, 1H), 4.02 (s, 3H) ppm; ^{13}C NMR (101 MHz, $CDCl_3$): δ 155.50, 148.66, 139.12, 129.57, 129.20, 126.54, 120.19, 119.72, 113.18, 107.56, 96.48, 57.77 ppm. HRMS (ESI) m/z calcd. for $C_{14}H_9BrF_3N_2$ [M+H]+: 340.9896, found 340.9901.

***3-bromo-2-(m-tolyl)-2H-indazole (2h)*[1]**

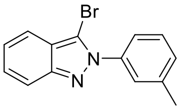

2h was obtained in 81% (46.33 mg) as a yellow oil;

^1H NMR (400 MHz, $CDCl_3$): δ 7.75 (d, J = 8.8 Hz, 1H), 7.58 (d, J = 8.8 Hz, 1H), 7.49-7.41 (m, 3H), 7.38-7.31 (m, 2H), 7.18 (t, J = 7.6 Hz, 1H), 2.47 (s, 3H) ppm; ^{13}C NMR (101 MHz, $CDCl_3$): δ 149.18, 139.44, 139.21, 130.13, 128.86, 127.66, 126.82, 123.28, 123.02, 122.92, 119.79, 118.24, 106.28, 21.47 ppm. MS:m/z 286 (M+).

3-bromo-2-(p-tolyl)-2H-indazole (2i)[1]

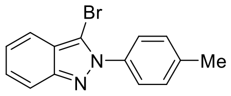

2i was obtained in 80% (45.76 mg) as a brown solid;

^1H NMR (400 MHz, $CDCl_3$): δ 7.76-7.72 (m, 1H), 7.58-7.51 (m, 3H), 7.35 (d, J = 8.4 Hz, 3H), 7.19-7.15 (m, 1H), 2.46 (s, 3H) ppm; ^{13}C NMR (101 MHz, $CDCl_3$): δ 149.19, 139.54, 136.89, 131.34, 129.76, 127.61, 126.05, 122.99, 121.99, 119.79, 118.25, 21.43 ppm. MS:m/z 286 (M+).

3-bromo-2-(4-methoxyphenyl)-2H-indazole (2j)[1]

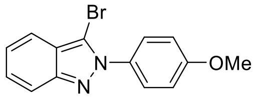

2j was obtained in 69% (41.68 mg) as a white solid;

^1H NMR (400 MHz, $CDCl_3$): δ 7.73 (d, J = 8.8 Hz, 1H), 7.58-7.56 (m, 3H), 7.37-7.33 (m, 1H), 7.19-7.15 (m, 1H), 7.05-7.03 (m, 2H), 3.88 (s, 3H) ppm; ^{13}C NMR (101 MHz, $CDCl_3$): δ 160.18, 149.07, 132.31, 131.26, 127.52, 122.94, 122.77, 119.74, 118.15, 114.26, 106.59, 60.52, 55.72 ppm. MS:m/z 302 (M+).

3-bromo-2-(2-methoxyphenyl)-2H-indazole (2k)

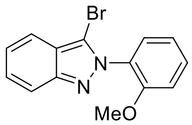

2k was obtained in 76% (45.91 mg) as a brown oil;

^1H NMR (400 MHz, $CDCl_3$): δ 7.75 (d, J = 8.8 Hz, 1H), 7.58 (d, J = 8.4 Hz, 1H), 7.53-7.48 (m, 1H), 7.42 (dd, J = 8.0, 1.6 Hz, 1H), 7.37-7.33 (m, 1H), 7.19-7.15 (m, 1H), 7.13-7.07 (m, 2H), 3.78 (s, 3H) ppm; ^{13}C NMR (101 MHz, $CDCl_3$): δ 154.66, 149.28, 131.54, 129.04, 128.04, 127.34, 122.69, 121.98, 120.69, 119.68, 118.29, 112.12, 109.16, 55.89 ppm. HRMS (ESI) m/z calcd. for $C_{14}H_{11}BrN_2O$ [M+H]+: 303.0128, found 303.0129.

3-bromo-2-(3-methoxyphenyl)-2H-indazole (2l)

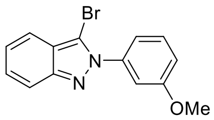

2l was obtained in 84% (50.74 mg) as a white solid;

^{1}H NMR (400 MHz, $CDCl_3$): δ 7.77 (d, *J* = 8.8 Hz, 1H), 7.59 (d, *J* = 8.4 Hz, 1H), 7.46 (t, *J* = 8.2 Hz, 1H), 7.38 (t, *J* = 7.6 Hz, 1H), 7.29-7.25 (m, 2H), 7.19 (t, *J* = 7.6 Hz, 1H), 7.07 (dd, *J* = 8.4, 2.0 Hz, 1H), 3.88 (s, 3H) ppm; ^{13}C NMR (101 MHz, $CDCl_3$): δ 160.02, 149.17, 140.22, 129.81, 127.71, 123.07, 122.95, 119.78, 118.44, 118.22, 115.48, 111.71, 106.26, 55.67 ppm. HRMS (ESI) m/z calcd. for $C_{14}H_{11}BrN_2O$ [M+H]+: 303.0128, found 303.0130.

3-bromo-2-(4-(trifluoromethoxy)phenyl)-2H-indazole (2m)

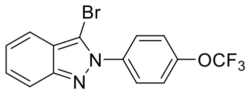

2m was obtained in 73% (51.97 mg) as a brown solid;

^{1}H NMR (400 MHz, $CDCl_3$) δ 7.73 (t, *J* = 8.0 Hz, 3H), 7.57 (d, *J* = 8.8 Hz, 1H), 7.42-7.35 (m, 3H), 7.21-7.17 (m, 1H) ppm; ^{13}C NMR (100 MHz, CDCl3): δ 149.48(d, *J* = 11 Hz), 137.63, 131.81, 128.10 (d, *J* = 5.0 Hz), 127.74 (d, *J* = 8.0 Hz), 123.42, 123.12, 121.99, 121.60, 119.82, 118.25, 106.37 ppm. HRMS (ESI) m/z calcd. for $C_{14}H_8BrF_3N_2O$ [M+H]+:356.9845, found 356.9849.

3-bromo-2-(4-fluorophenyl)-2H-indazole (2n)[1]

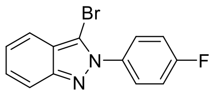

2n was obtained in 85% (49.30 mg) as a yellow solid;

^{1}H NMR (400 MHz, $CDCl_3$) δ 7.74 (d, *J* = 8.8 Hz, 1H), 7.69-7.64 (m, 2H), 7.61-7.56 (m, 1H), 7.43-7.36 (m, 1H), 7.26 (t, *J* = 8.6 Hz, 2 H), 7.22-7.18 (m, 1H) ppm; ^{13}C NMR (101 MHz, $CDCl_3$) ppm: δ 164.12, 161.64, 149.34, 128.20 (d, *J* = 8.8 Hz), 127.88, 123.25, 122.97, 119.78, 118.25, 116.33, 106.47 ppm. MS:m/z 290 (M+).

3-bromo-2-(4-chlorophenyl)-2H-indazole (2o)[1]

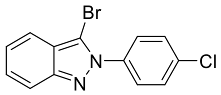

2o was obtained in 80% (48.95 mg) as a yellow solid;

^{1}H NMR (400 MHz, $CDCl_3$): δ 7.729 (d, *J* = 8.8 Hz, 1H), 7.64 (d, *J* = 8.8 Hz, 2H), 7.57 (d, *J* = 8.4 Hz, 1H), 7.53 (d, *J* = 8.8 Hz, 2H), 7.373 (t, *J* = 8.2 Hz, 1H), 7.20-7.17 (m, 1H) ppm; ^{13}C NMR (101 MHz,

CDCl3): δ 149.42, 137.79, 135.35, 129.42, 127.99, 127.47, 123.34, 119.81, 118.25, 106.29 ppm. MS:m/z 306 (M+).

3-bromo-2-(4-iodophenyl)-2H-indazole (2p)

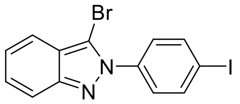

2p was obtained in 82% (65.25 mg) as a brown solid;

^{1}H NMR (400 MHz, $CDCl_3$): δ 7.88 (d, *J* = 8.4 Hz, 2H), 7.72 (d, *J* = 8.8 Hz, 1H), 7.56 (d, *J* = 7.6 Hz,1H), 7.46 (d, *J* = 8.4 Hz, 2H), 7.36 (t, *J* = 7.6 Hz, 1H), 7.18 (t, *J* = 7.6Hz, 1H) ppm; ^{13}C NMR (101 MHz, $CDCl_3$): δ 149.46, 139.00, 138.36, 128.00, 127.84, 123.34, 123.18, 119.81, 118.27, 106.12, 94.99 ppm. HRMS (ESI) m/z calcd. for $C_{13}H_8BrIN_2$ [M+H]+: 398.8988, found 398.8992.

***3-bromo-2-(3-chlorophenyl)-2H-indazole (2q)*[1]**

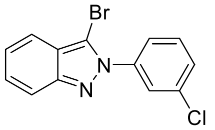

2q was obtained in 77% (47.12 mg) as a yellow solid;

^{1}H NMR (400 MHz, $CDCl_3$) δ 7.73 (d, *J* = 8.8 Hz, 2H), 7.62-7.59 (m, 1H), 7.57 (d, *J* = 8.8 Hz, 1H), 7.49-7.47 (m, 2H), 7.39-7.35 (m, 1H), 7.18 (t, *J* = 7.6 Hz, 1H) ppm; ^{13}C NMR (101 MHz, $CDCl_3$): δ 149.41, 140.20, 134.87, 130.12, 129.47, 128.05, 126.52, 124.36, 123.39, 123.12, 119.80, 118.26, 106.25 ppm. MS:m/z 306 (M+).

3-bromo-2-(3-bromophenyl)-2H-indazole (2r)

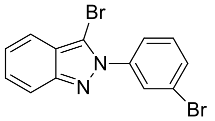

2r was obtained in 80% (55.99 mg) as a white solid;

^{1}H NMR (400 MHz, $CDCl_3$): δ 7.89 (t, *J* = 2.0 Hz, 1H), 7.72 (d, *J* = 8.8 Hz, 1H), 7.66-7.62 (m, 2H), 7.56 (d, *J* = 8.4 Hz, 1H), 7.41 (d, *J* = 8.0 Hz, 1H), 7.38-7.34 (m, 1H), 7.17 (t, *J* = 7.6 Hz, 1H) ppm; ^{13}C NMR (101 MHz, $CDCl_3$): δ 149.40, 140.25, 132.34, 130.31, 129.31, 128.02, 124.78, 123.36, 123.09, 122.55, 119.77, 118.24, 106.21 ppm. HRMS (ESI) m/z calcd. for $C_{13}H_8Br_2N_2$ [M+H]+: 350.9127, found 350.9125.

3-bromo-2-(3-iodophenyl)-2H-indazole (2s)

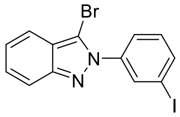

2s was obtained in 88% (70.03 mg) as a yellow solid;

^{1}H NMR (400 MHz, $CDCl_3$): δ 8.09 (s, 1H), 7.84 (d, *J* = 7.6 Hz, 1H), 7.74 (d, *J* = 8.8 Hz, 1H), 7.69 (d, *J* = 8.0 Hz, 1H), 7.57 (d, *J* = 8.4 Hz, 1H), 7.38 (t, *J* = 7.6 Hz, 1H), 7.27 (t, *J* = 8.0 Hz, 1H), 7.18 (t, *J* = 7.6 Hz, 1H) ppm; ^{13}C NMR (101 MHz, $CDCl_3$): δ 149.35, 140.05, 138.26, 134.97, 130.39, 127.99, 125.42, 123.33, 123.05, 119.76, 118.21, 106.22, 93.73 ppm. HRMS (ESI) m/z calcd. for $C_{13}H_8BrIN_2$ [M+H]+: 398.8988, found 398.8986.

methyl 3-(3-bromo-2H-indazol-2-yl)benzoate (2t)

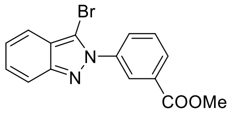

2t was obtained in 77% (50.82 mg) as a red solid;

^{1}H NMR (400 MHz, $CDCl_3$): δ 8.38 (t, *J* = 1.8 Hz, 1H), 8.18 (dt, *J* = 7.6 Hz, 1.2 Hz, 1H), 7.90-7.87 (m, 1H), 7.73 (d, *J* = 8.8 Hz, 1H), 7.62 (t, *J* = 8.0 Hz, 1H), 7.56 (d, *J* = 8.4 Hz, 1H), 7.38-7.34 (m, 1H), 7.19-7.15 (m, 1H) ppm; ^{13}C NMR (101 MHz, $CDCl_3$): δ 165.88, 149.42, 139.45, 131.37, 130.41, 130.26, 129.33, 127.96, 127.27, 123.31, 123.07, 119.78, 118.24, 106.29, 52.59 ppm. HRMS (ESI) m/z calcd. for $C_{15}H_{11}BrN_2O_2$ [M+H]+: 331.0077, found 331.0075.

methyl 4-(3-bromo-2H-indazol-2-yl)benzoate (2u)

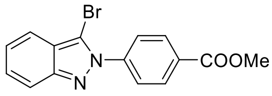

2u was obtained in 80% (52.80 mg) as a white solid;

^{1}H NMR (400 MHz, $CDCl_3$): δ 8.22 (d, *J* = 8.8 Hz, 2H), 7.81 (d, *J* = 8.4 Hz, 2H), 7.72 (d, *J* = 8.8 Hz, 1 H), 7.56 (d, *J* = 8.4 Hz, 1H), 7.38-7.34 (m, 1H), 7.19-7.16 (m, 1H), 3.96 (s, 3H) ppm; ^{13}C NMR (101 MHz, $CDCl_3$): δ 166.17, 149.57, 142.76, 131.89, 130.58, 128.14, 126.02, 123.46, 122.00, 119.84, 118.30, 106.13, 52.59 ppm. HRMS (ESI) m/z calcd. for $C_{15}H_{11}BrN_2O_2$ [M+H]+: 331.0077, found 331.0076.

3-bromo-2-(3-(trifluoromethyl)phenyl)-2H-indazole (2v)

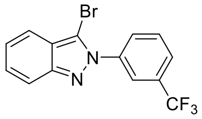

2v was obtained in 68% (46.24 mg) as a brown solid;

^{1}H NMR (400 MHz, $CDCl_3$) δ 8.03 (s, 1H), 7.91 (d, *J* = 8.0 Hz,1H), 7.78-7.73 (m, 2H), 7.66 (t, *J* = 7.8 Hz, 1H), 7.56 (d, *J* = 8.8 Hz, 1H), 7.39-7.35 (m, 1H), 7.20-7.16 (m, 1H) ppm; ^{13}C NMR (101 MHz, $CDCl_3$): δ 149.57, 139.69, 131.84 (q, *J* = 49.5 Hz), 129.82, 129.36, 128.18, 125.94 (q, *J* = 5.5 Hz), 124.85, 123.51, 123.32 (q, *J* = 6.0 Hz), 119.80, 118.26, 106.25 ppm. HRMS (ESI) m/z calcd. for $C_{14}H_8BrF_3N2$ [M+H]+: 340.9896, found 340.9892.

3-bromo-2-cyclohexyl-2H-indazole (2w)

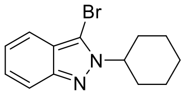

2w was obtained in 77% (42.82 mg) as a white solid;

^{1}H NMR (400 MHz, $CDCl_3$): δ 7.72 (d, *J* = 8.8 Hz, 1H), 7.54 (d, *J* = 8.4 Hz, 1H), 7.33-7.28 (m, 1H), 7.15-7.11 (m, 1H), 4.66-4.59 (m, 1H), 2.12-2.07 (m, 4H), 1.98 (d, *J* = 13.6 Hz, 2H), 1.79 (d, *J* = 12.8 Hz, 2H), 1.57-1.45 (m, 2H), 1.43-1.35 (m, 1H) ppm; ^{13}C NMR (101 MHz, $CDCl_3$): δ 148.27, 126.64, 122.24, 121.59, 119.31, 118.00, 104.90, 60.35, 32.85, 26.68, 25.25 ppm. HRMS (ESI) m/z calcd. for $C_{13}H_{15}BrN_2$ [M+H]+: 279.0491, found 279.0497.

3-bromo-5-iodo-2-methyl-2H-indazole (2x)

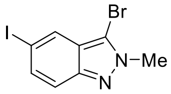

2x was obtained in 75% (67.39 mg) as a white solid;

^{1}H NMR (400 MHz, $CDCl_3$): δ 7.89 (s, 1H), 7.50 (d, *J* = 9.1 Hz, 1H), 7.41 (d, *J* = 9.0 Hz, 1H), 4.17 (s, 3H). ppm; ^{13}C NMR (101 MHz, $CDCl_3$): δ 135.57, 128.32, 124.30, 119.63, 105.44, 86.47, 39.08 ppm. HRMS (ESI) m/z calcd. for $C_8H_6BrIN_2$ [M+H]+: 336.8832, found 336.8835.

1-benzyl-3-bromo-1H-indazole (2y)

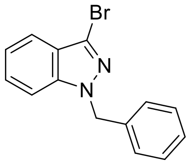

2y was obtained in 93% (67.39 mg) as a white solid;

^{1}H NMR (400 MHz, $CDCl_3$) δ 8.09 (s, 1H), 7.78 (d, *J* = 8.1 Hz, 1H), 7.37 (d, *J* = 5.9 Hz, 2H), 7.3-7.3 (m, 3H), 7.23 (d, *J* = 6.9 Hz, 2H), 7.17 (t, *J* = 6.9 Hz, 1H), 5.6 (s, 2H) ppm; ^{13}C NMR (101 MHz, $CDCl_3$) δ 139.66, 137.00, 133.48, 128.82, 127.83, 127.27, 126.49, 124.47, 121.25, 120.75, 109.39, 53.07 ppm. HRMS (ESI) m/z calcd. for $C_{14}H_{13}BrN_2$ [M+H]+: 289.0335, found 289.0338.

3-chloro-2-phenyl-2H-indazole (2z)[1]

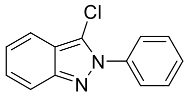

2z was obtained in 83% (37.96 mg) as a brown solid;

^{1}H NMR (400 MHz, $CDCl_3$) δ 7.71 (t, J = 8.1 Hz, 3H), 7.63 (d, J = 8.5 Hz, 1H), 7.55 (m, 3H), 7.41 - 7.31 (t, J = 8.1 Hz, 1H), 7.21 - 7.13 (t, J = 8.1 Hz, 1H) ppm; ^{13}C NMR (101 MHz, $CDCl_3$) δ 148.68, 138.61, 129.27, 127.72, 125.85, 122.92, 120.02, 119.66, 119.13, 118.33 ppm. MS:m/z 228 (M+).

3,5-bis(4-methoxyphenyl)-2-phenyl-2H-indazole (4)

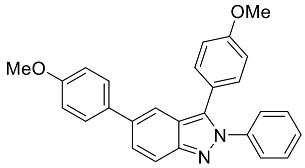

4 was obtained in 80% (65.04 mg) as a white solid;

^{1}H NMR (400 MHz, $CDCl_3$): δ 7.85 (d, J = 8.8 Hz, 1H), 7.80 (s, 1H), 7.64 (dd, J = 8.8 Hz, 1.6 Hz, 1H), 7.57 (d, J = 8.8 Hz, 2H), 7.48-7.46 (m, 2H), 7.43-7.38 (m, 3H), 7.32 (d, J = 8.8 Hz, 2H), 6.99 (d, J = 8.8 Hz, 2H), 6.94 (d, J = 8.8 Hz, 2H), 3.85 (s, 6H) ppm; ^{13}C NMR (101 MHz, $CDCl_3$): δ 159.83, 159.05, 148.15, 140.22, 135.97, 135.37, 134.37, 131.12, 129.15, 128.39, 128.35, 127.97, 126.09, 122.18, 122.14, 117.96, 117.58, 114.52, 114.32, 55.49, 55.43 ppm. HRMS (ESI) m/z calcd. for $C_{27}H_{22}N_2O_2$ [M+H]+: 407.1754, found 407.1757.

3-bromo-2H-indazole (3a)[1]

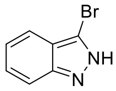

3a was obtained in 65% (25.48 mg) as a white solid;

^{1}H NMR (400 MHz, $CDCl_3$): δ 12.20 (s, 1H), 7.70 (dd, J = 8.2, 4.5 Hz, 2H), 7.58 - 7.37 (m, 1H), 7.37 - 7.16 (m, 1H) ppm; ^{13}C NMR (101 MHz, $CDCl_3$): δ 141.43, 128.27, 123.07, 122.55, 121.97, 120.21, 110.83 ppm. MS:m/z 196 (M+).

References

1. Liu X, Wu Z, Feng C, et al. Catalyst-and Oxidant-Free Electrochemical Halogenation Reactions of 2H-Indazoles with NaX (X= Cl, Br). European Journal of Organic Chemistry, 2022, 2022(17): e202200262.

4. ^{1}H NMR and ^{13}C NMR spectra of products

^{1}H NMR spectra for **2a** 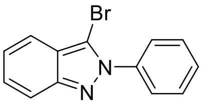

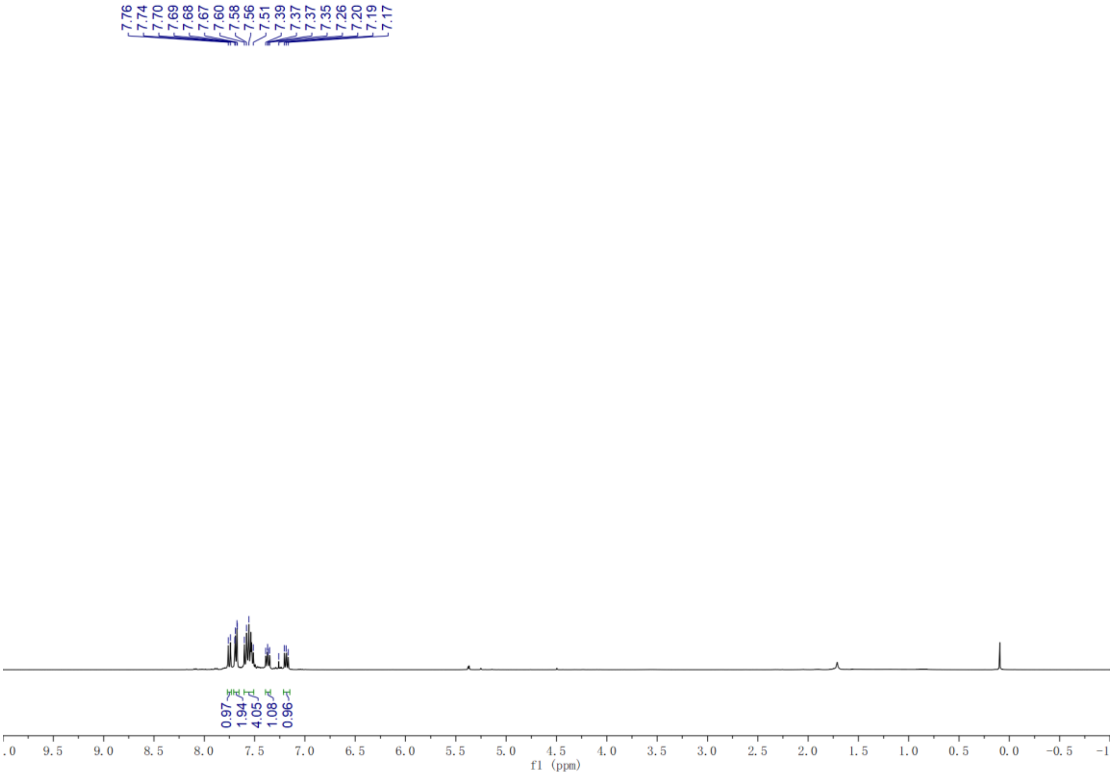

^{13}C NMR spectra for **2a**

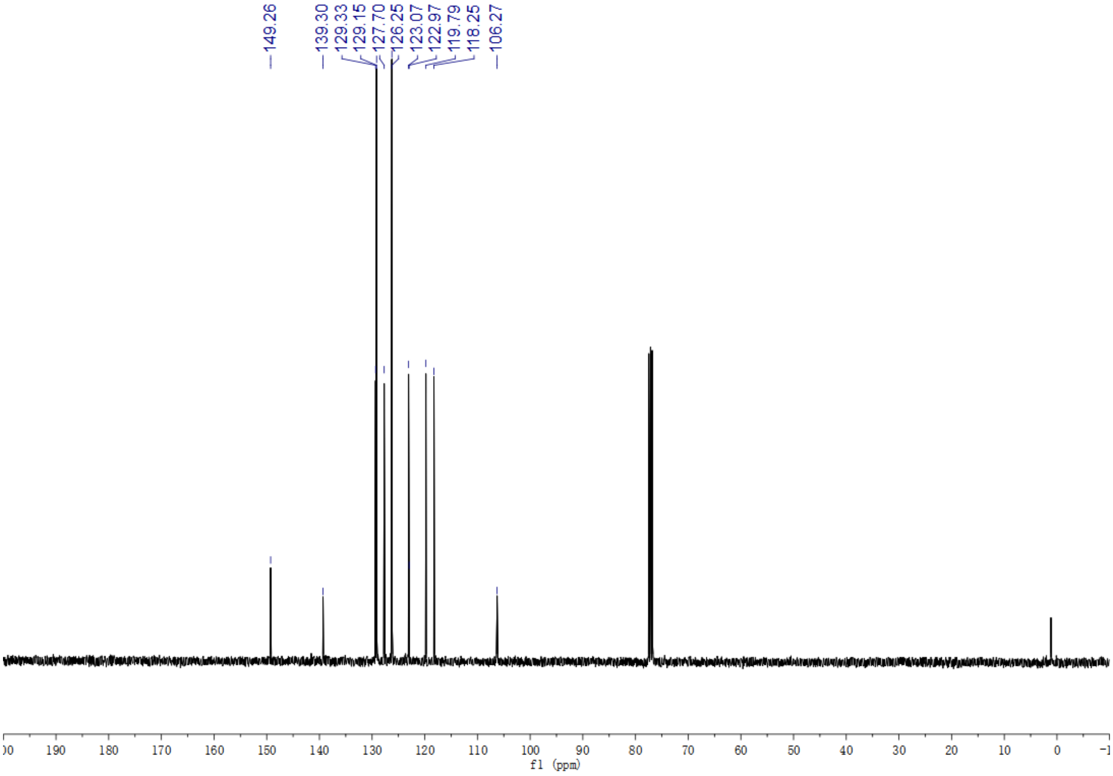

^{1}H NMR spectra for **2b** 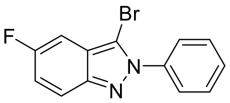

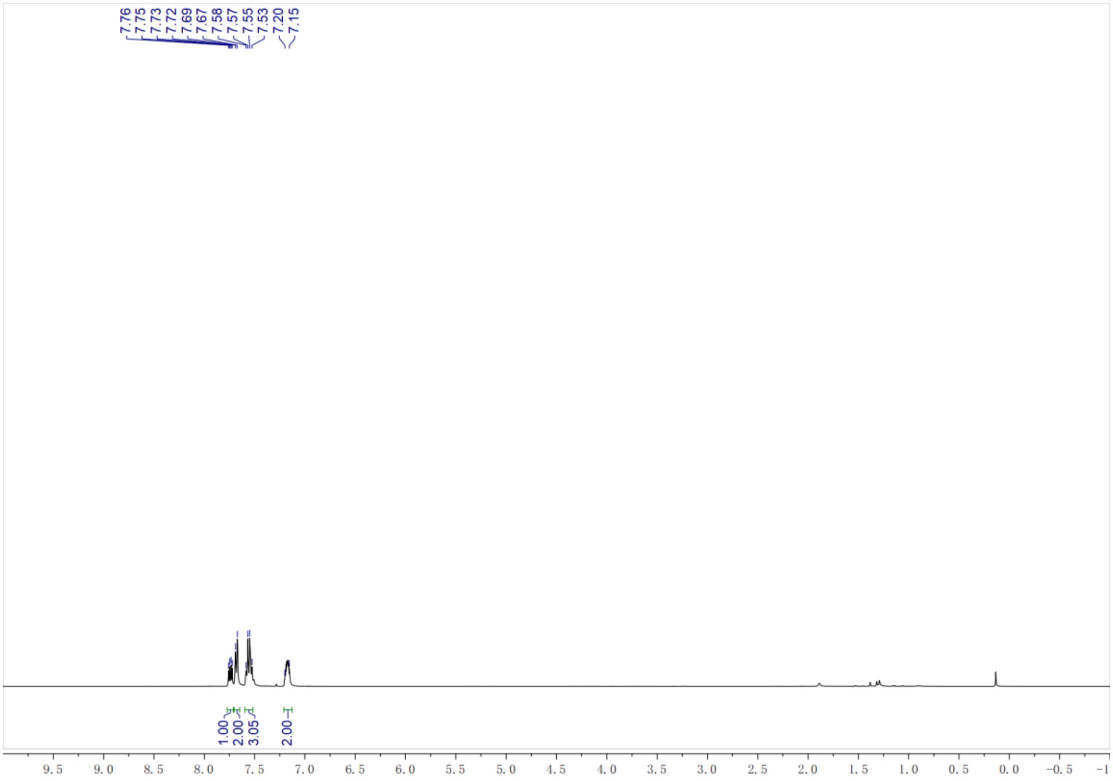

^{13}C NMR spectra for **2b**

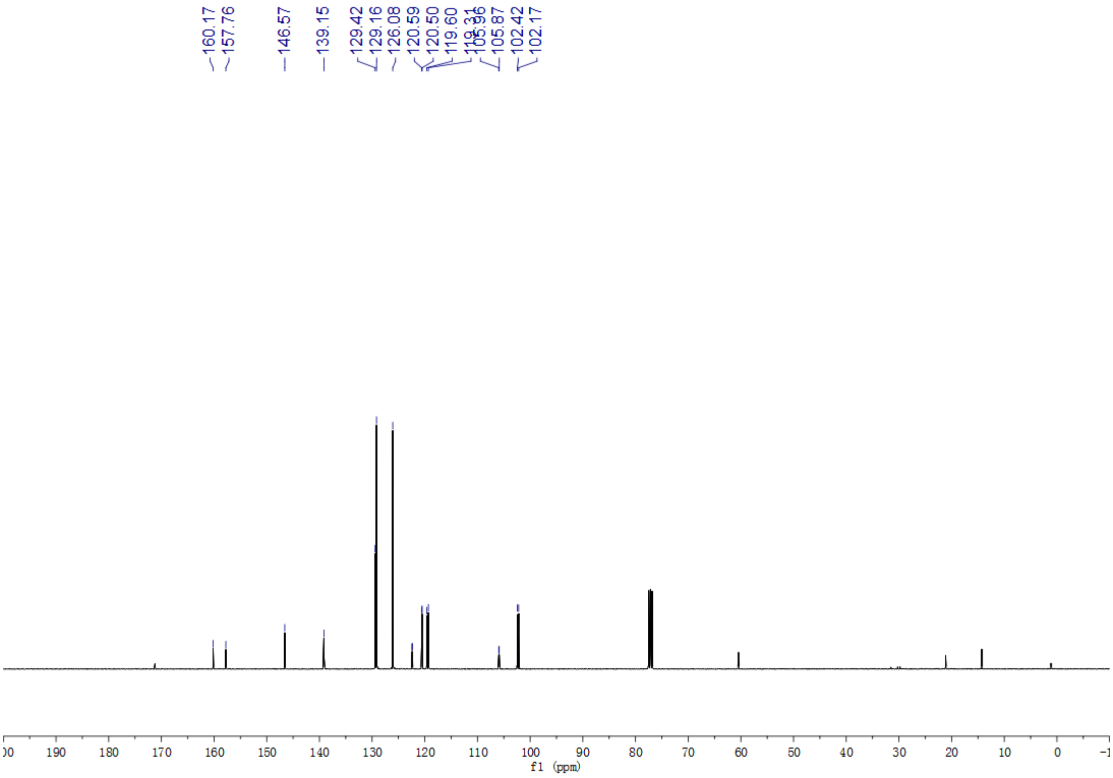

^{1}H NMR spectra for **2c**

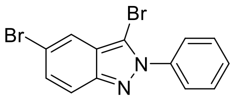

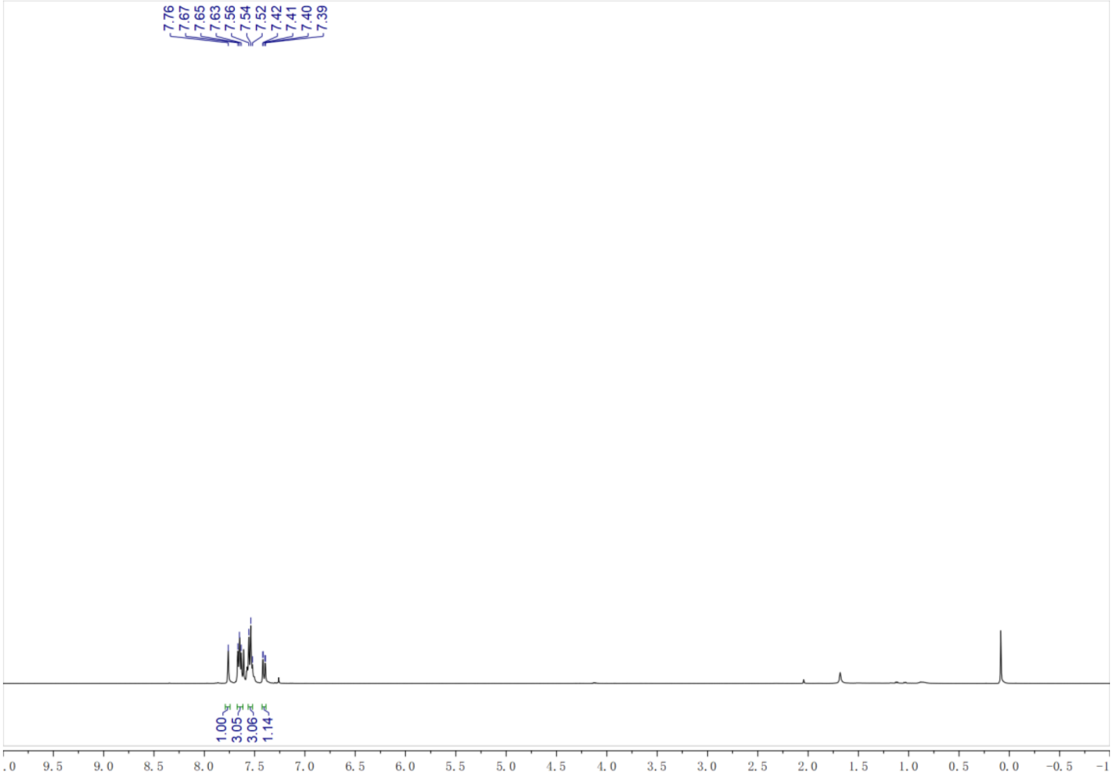

^{13}C NMR spectra for **2c**

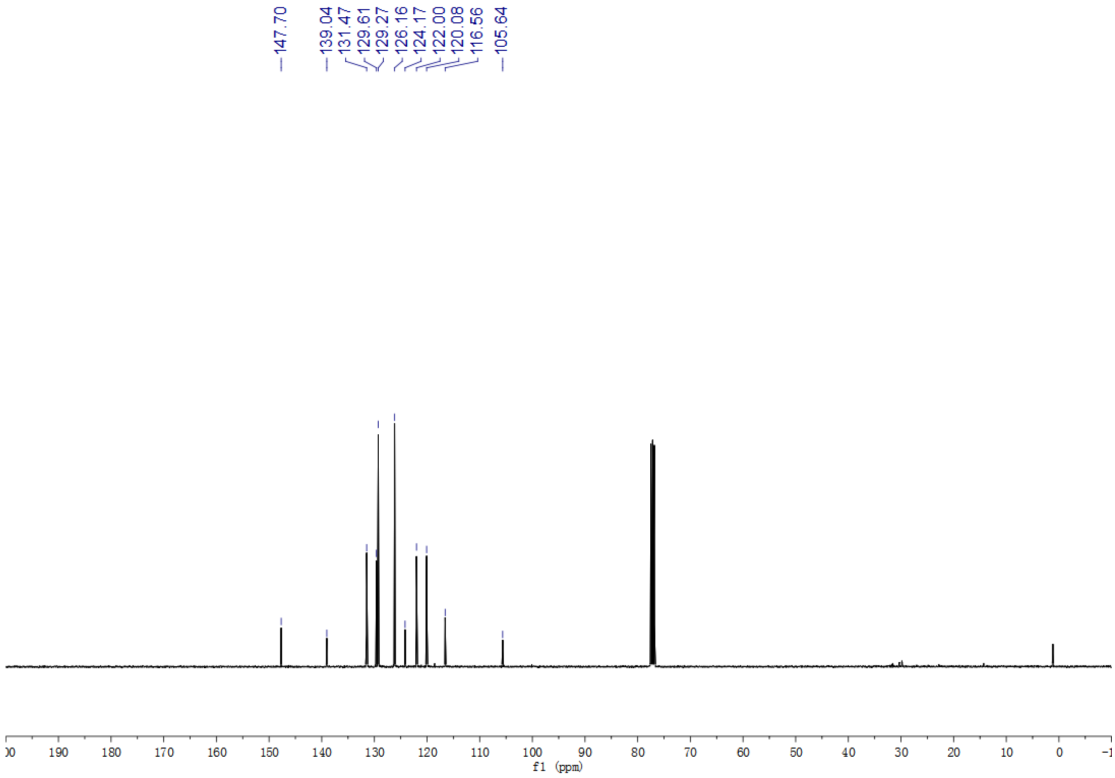

^{1}H NMR spectra for **2d** 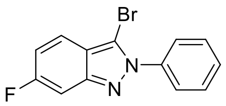

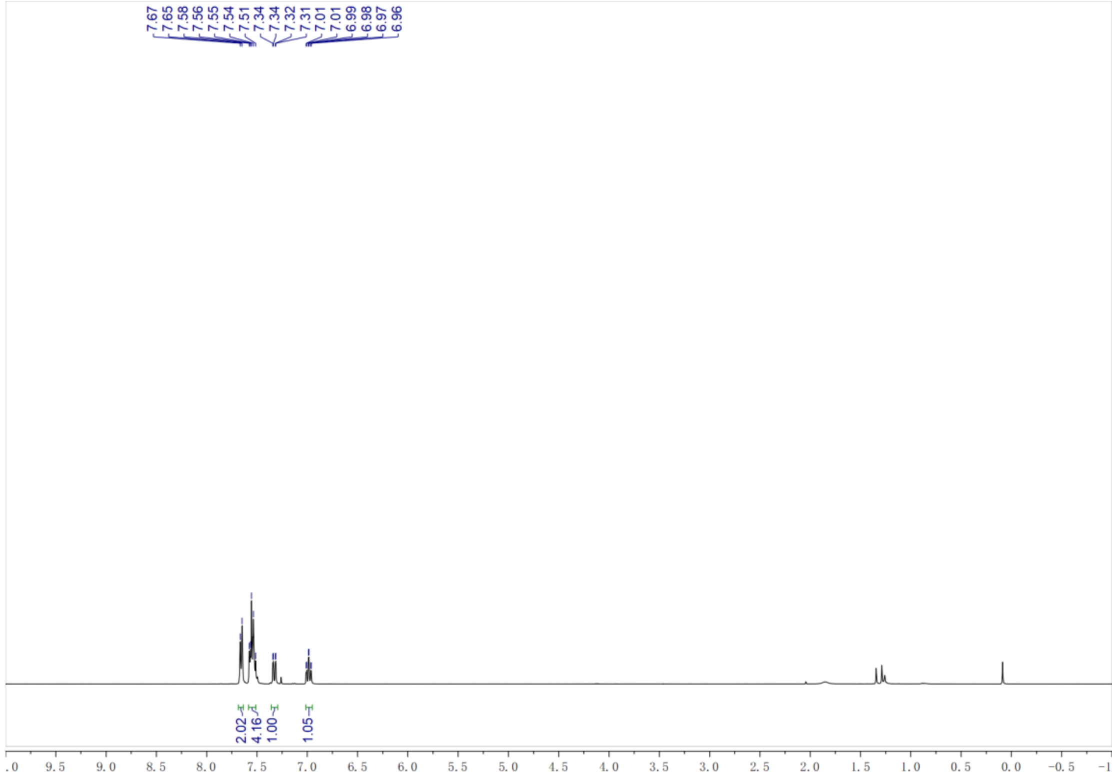

^{13}C NMR spectra for **2d**

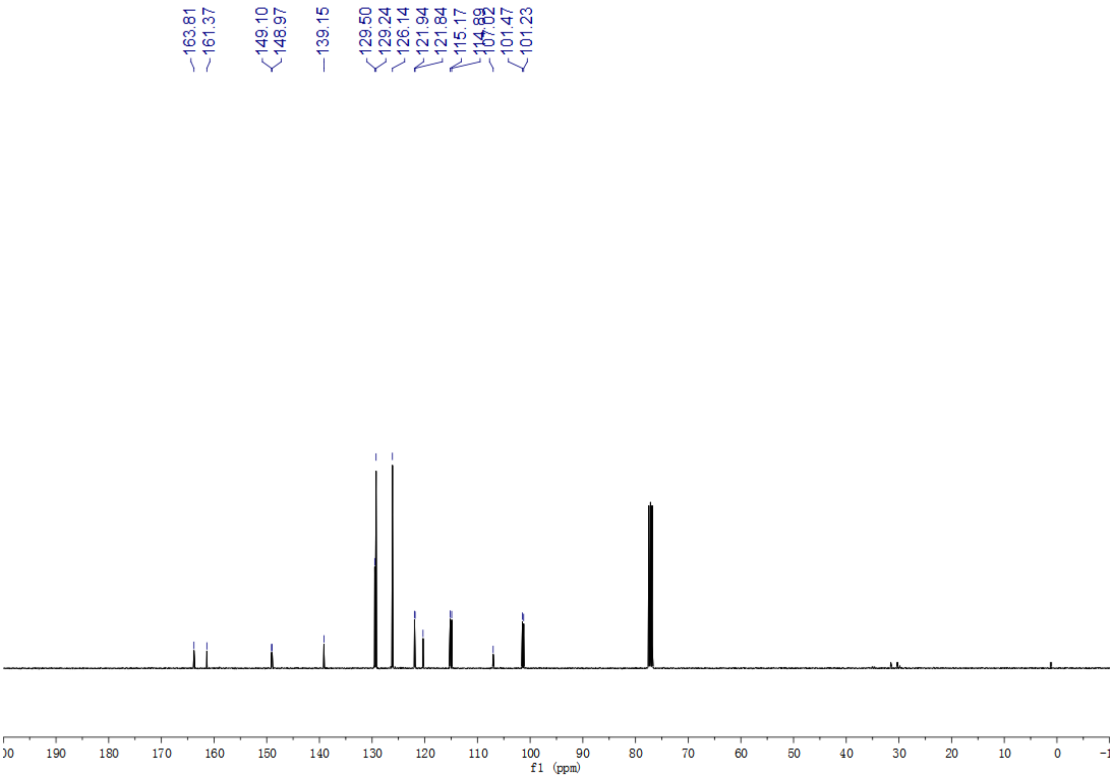

^{1}H NMR spectra for **2e**

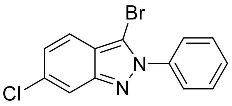

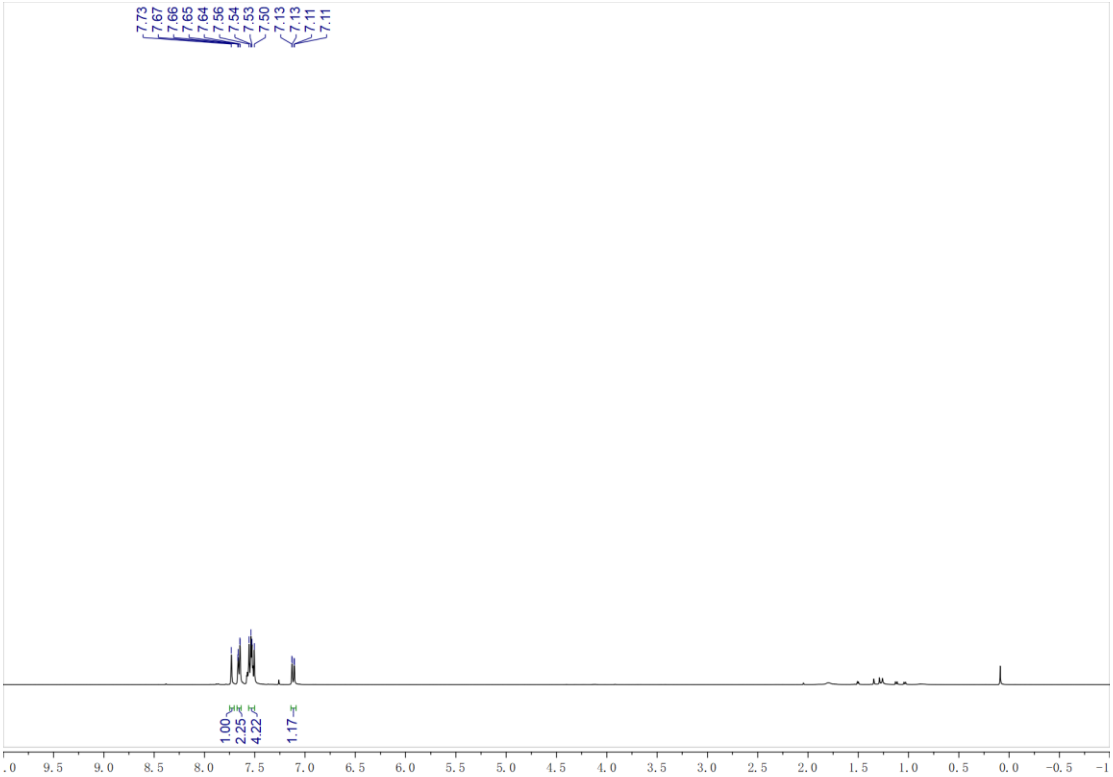

^{13}C NMR spectra for **2e**

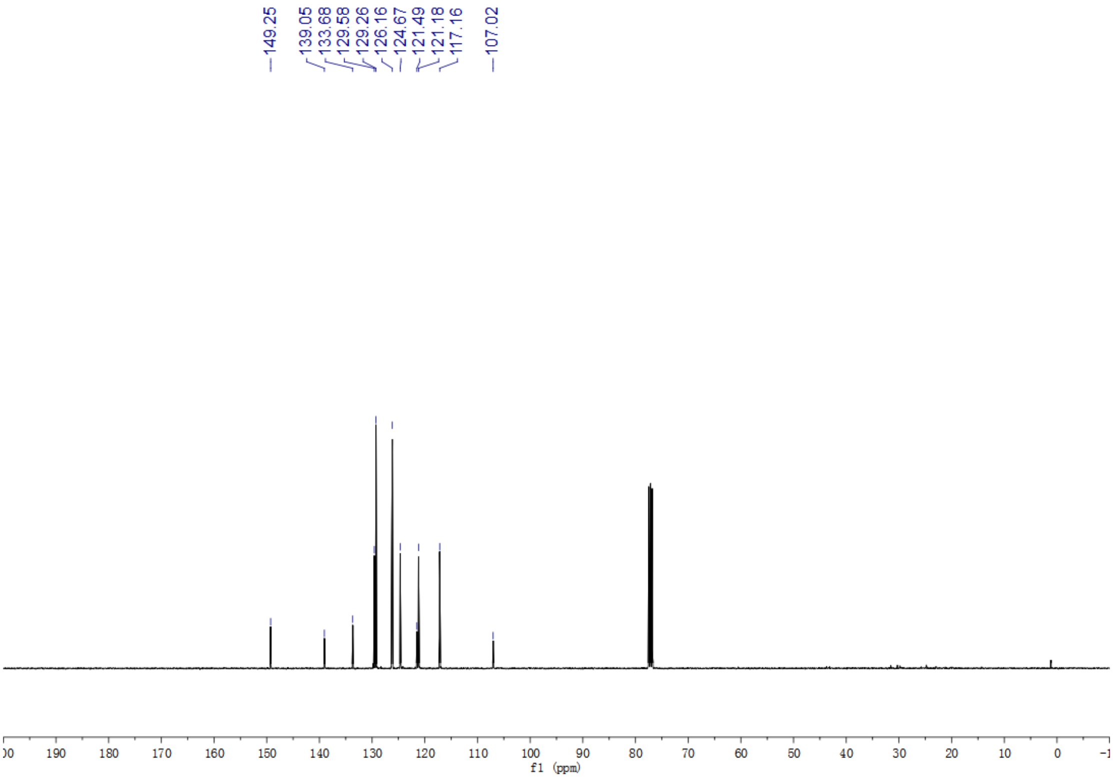

^{1}H NMR spectra for **2f**

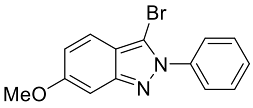

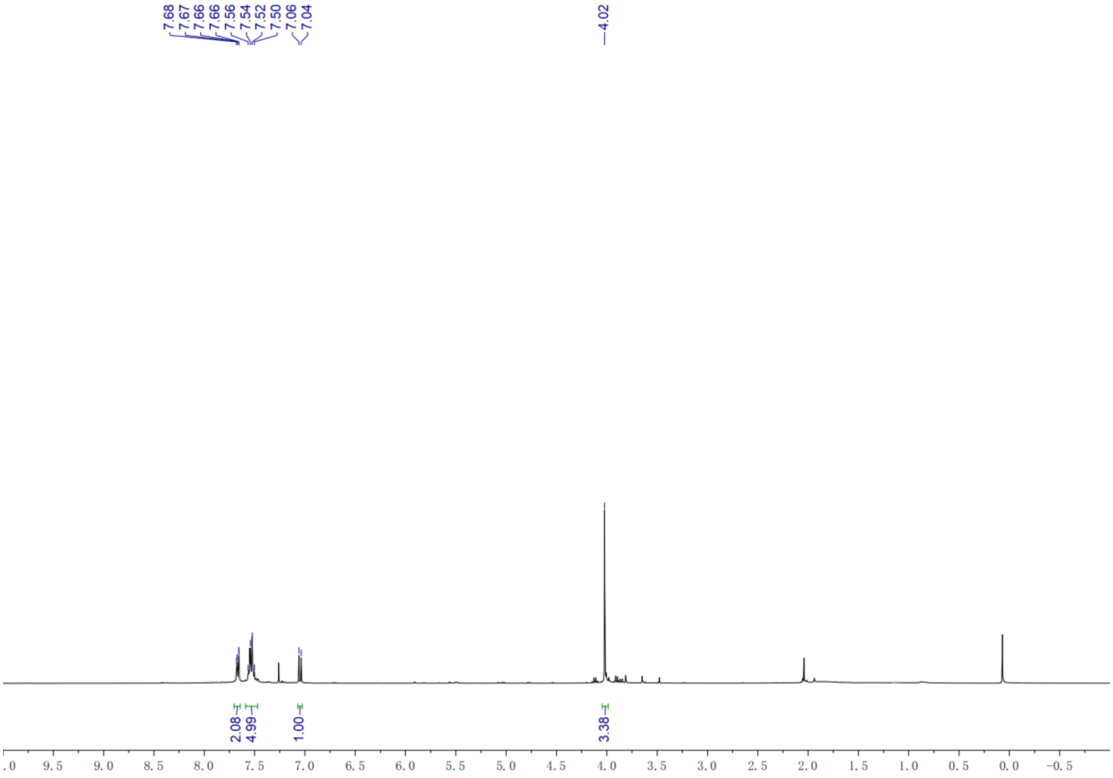

^{13}C NMR spectra for **2f**

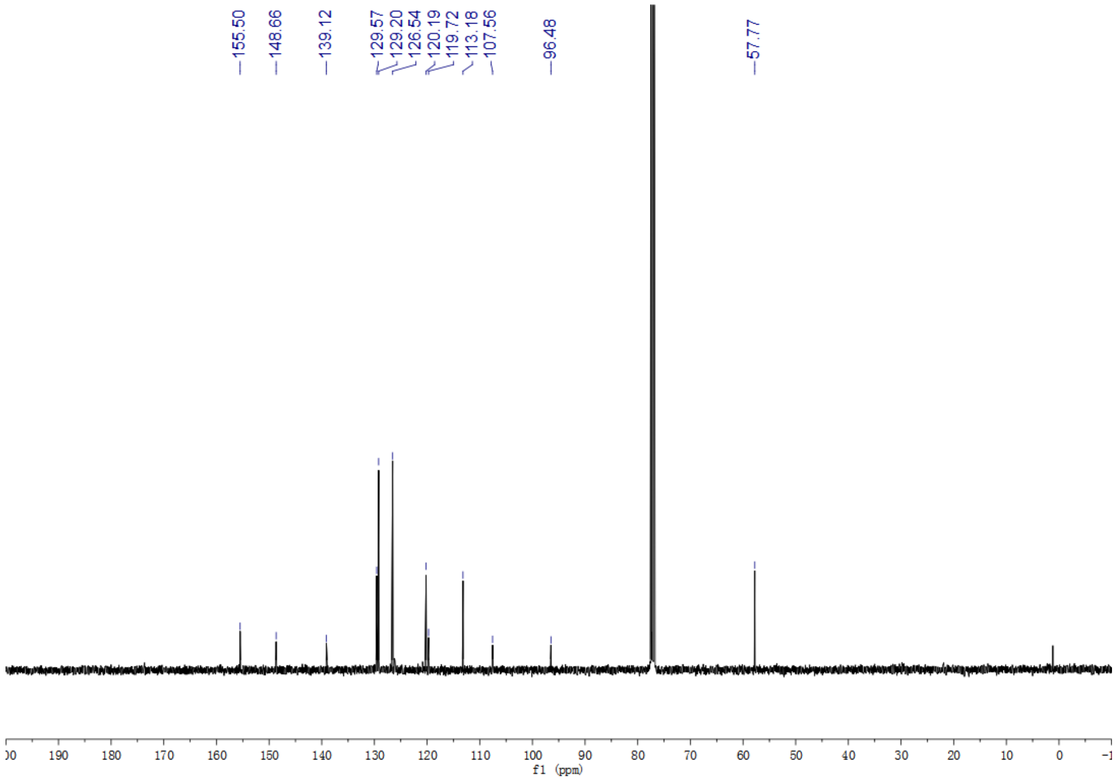

^{1}H NMR spectra for **2g**

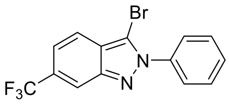

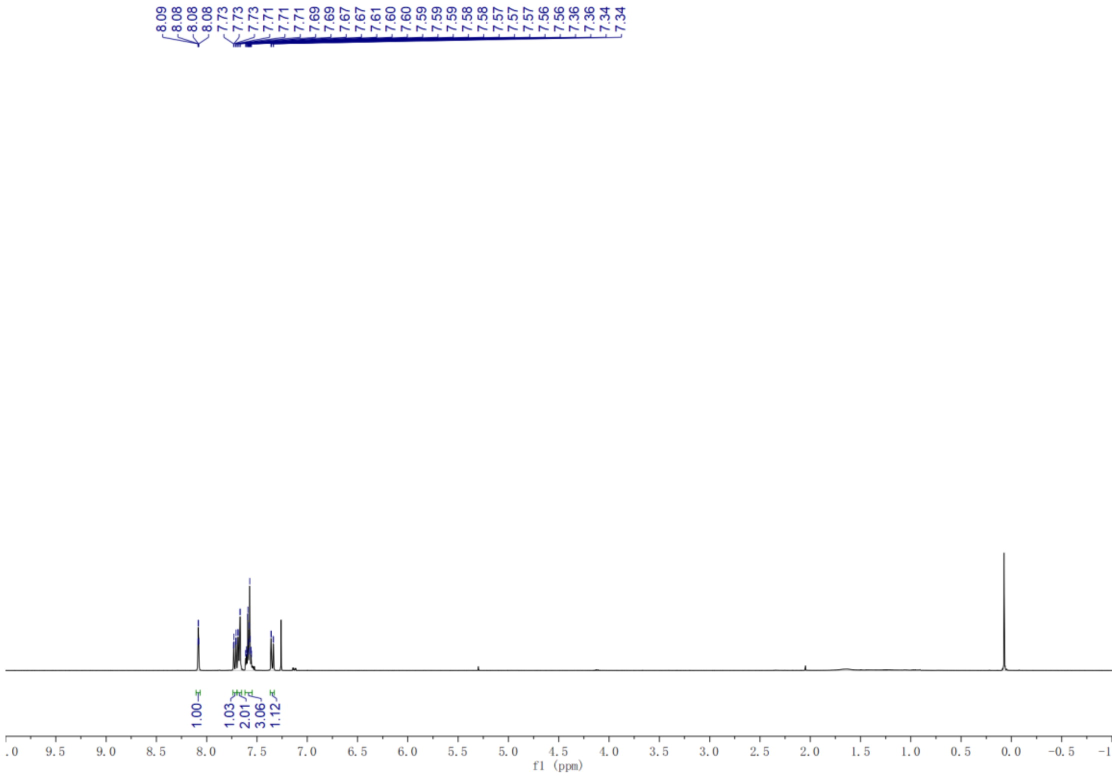

^{13}C NMR spectra for **2g**

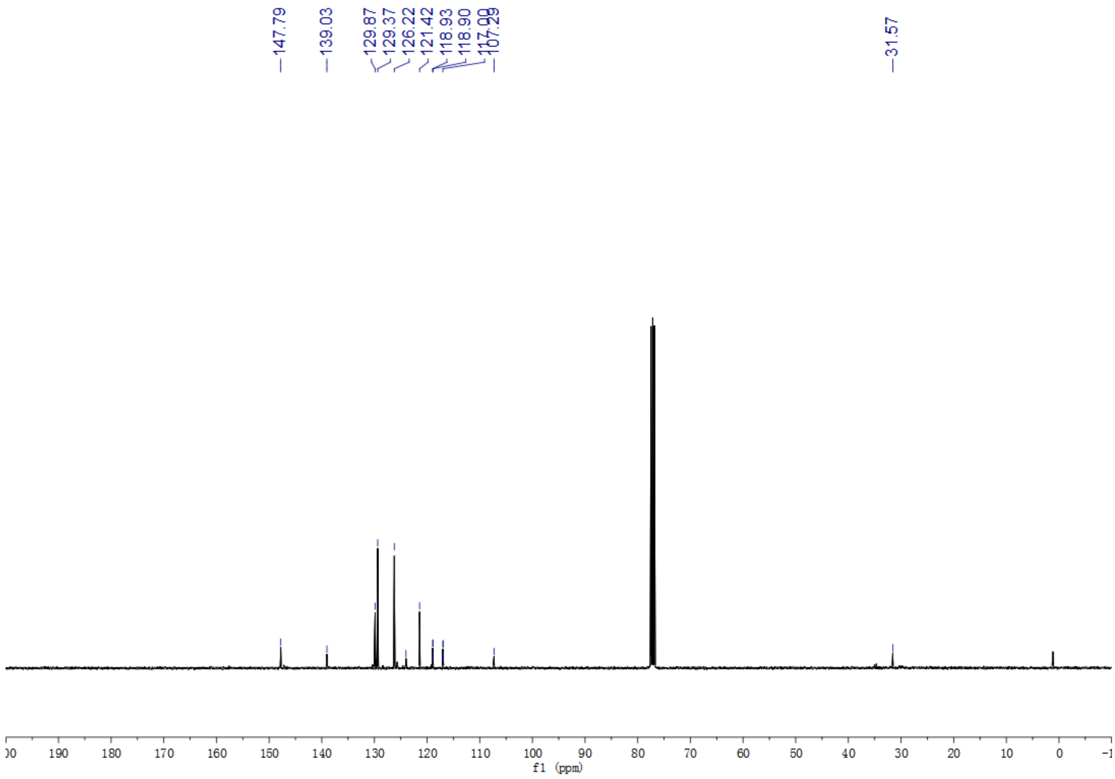

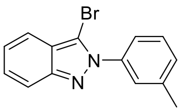

^{1}H NMR spectra for **2h**

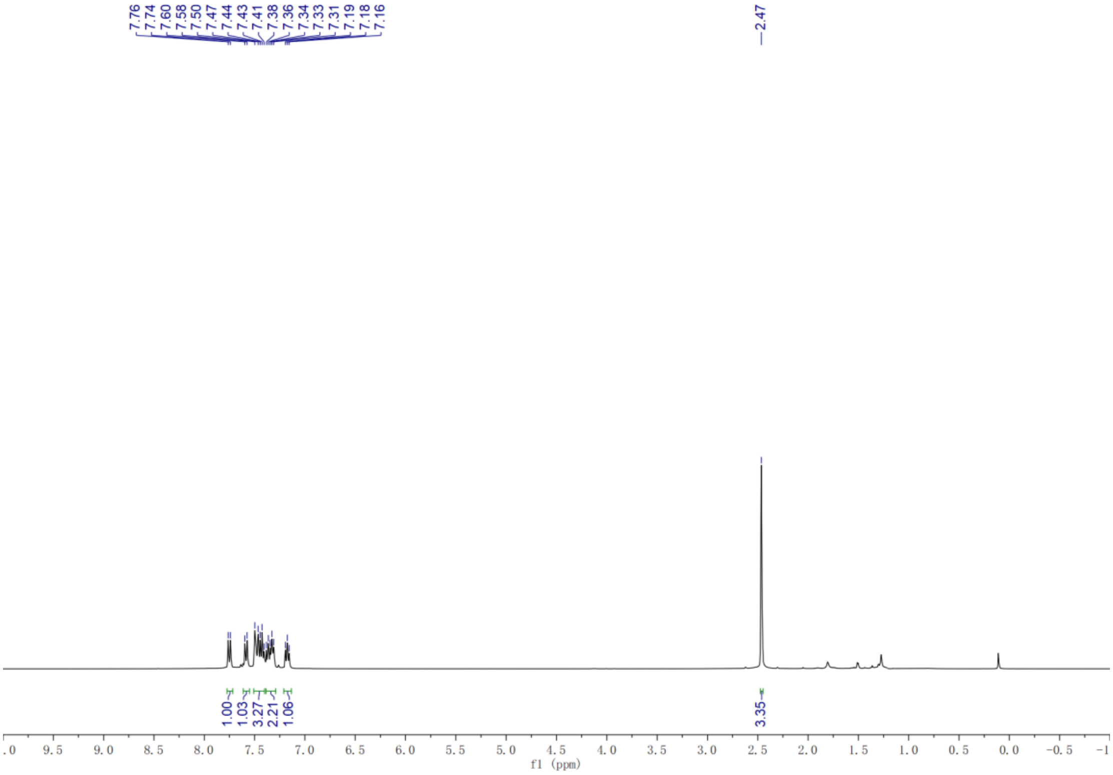

^{13}C NMR spectra for **2h**

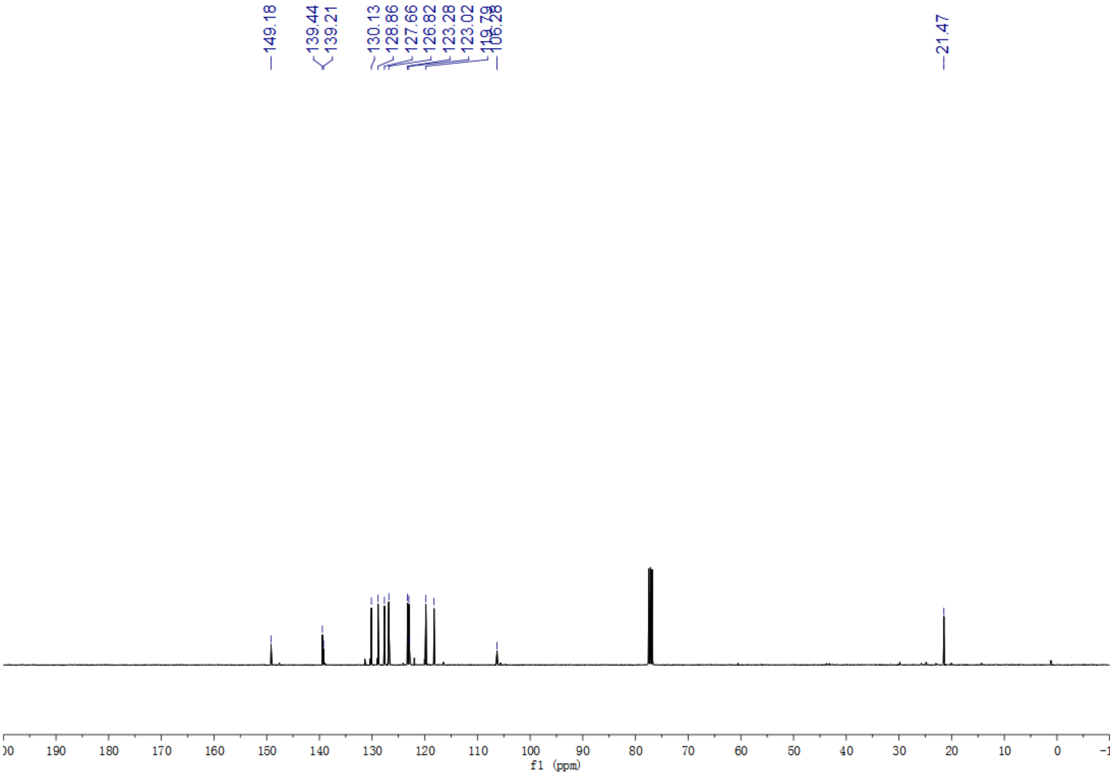

^{1}H NMR spectra for **2i**

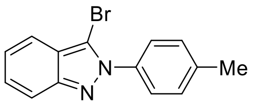

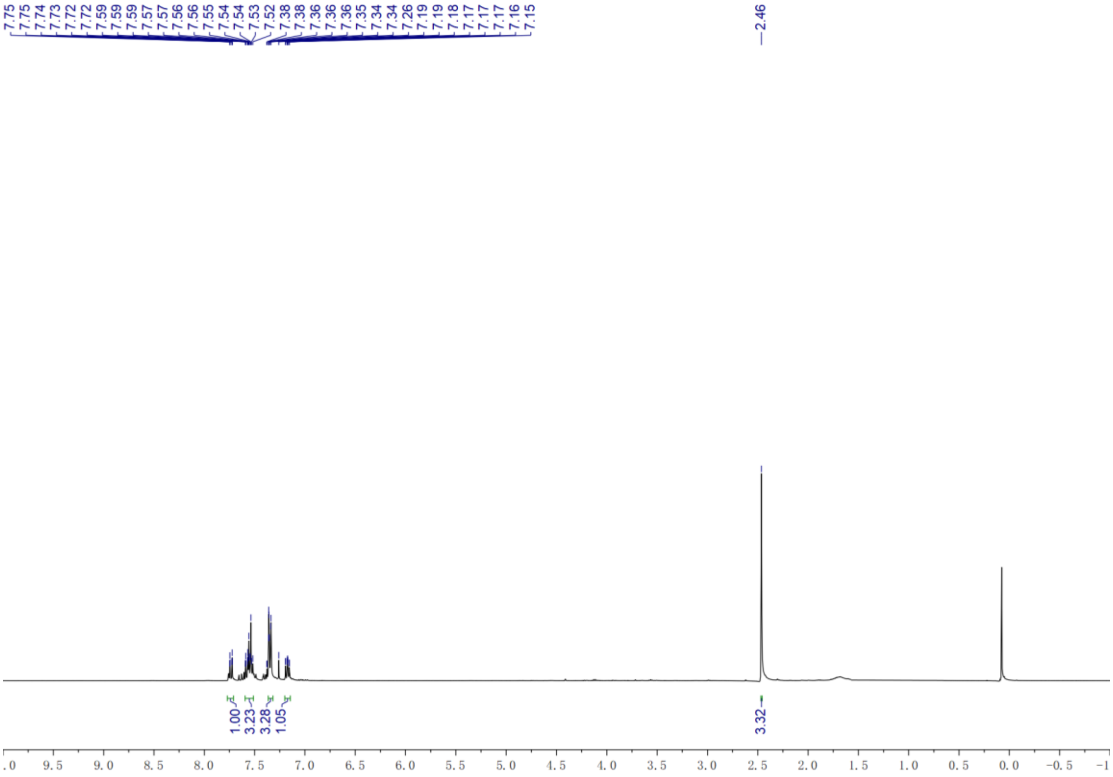

^{1}H NMR spectra for **2i**

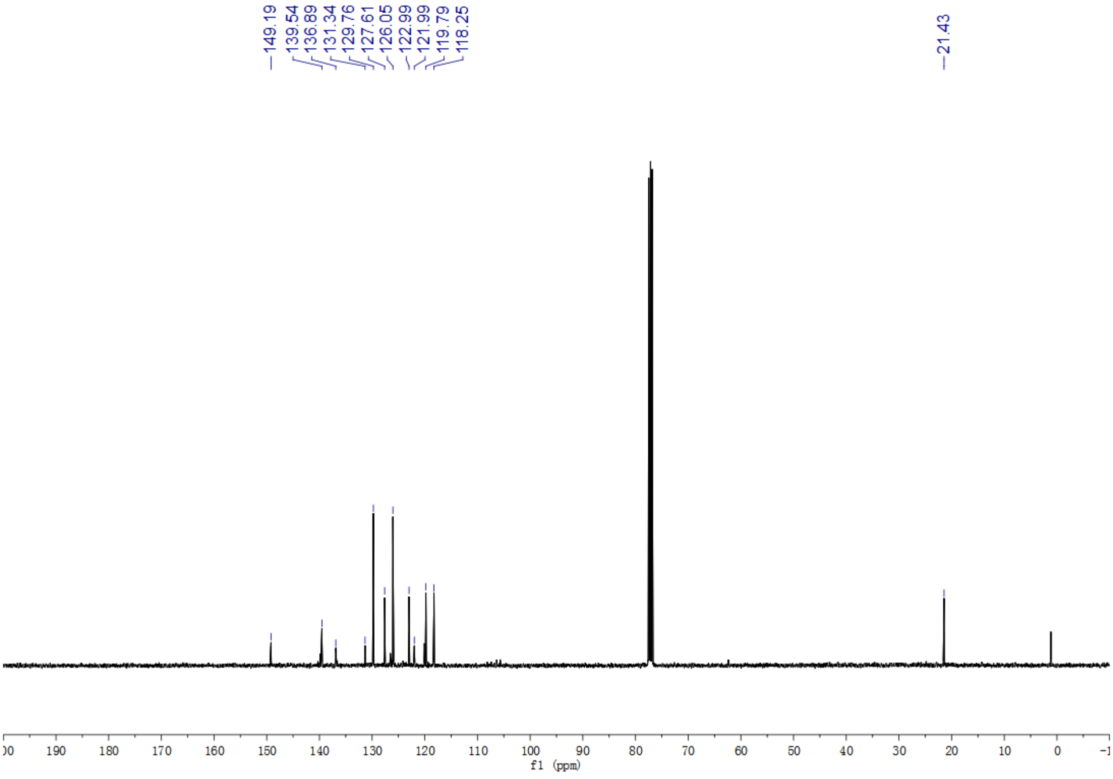

^{13}C NMR spectra for **2j**

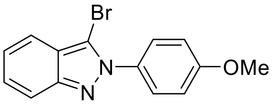

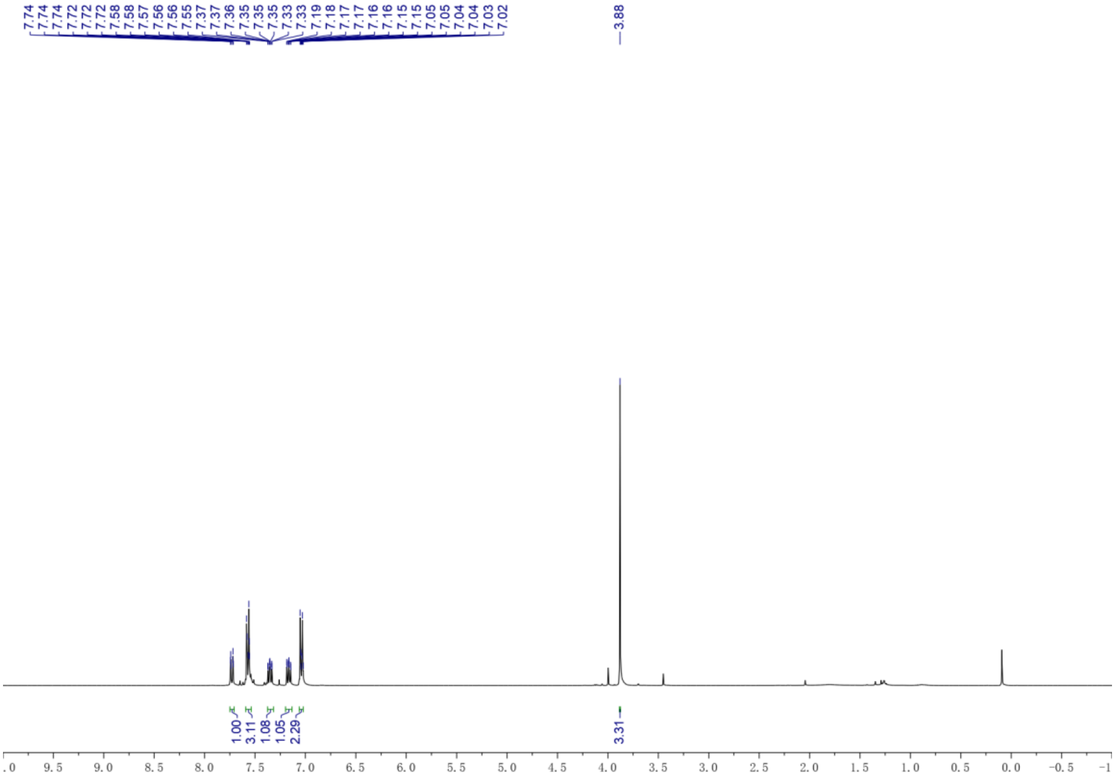

^{13}C NMR spectra for **2j**

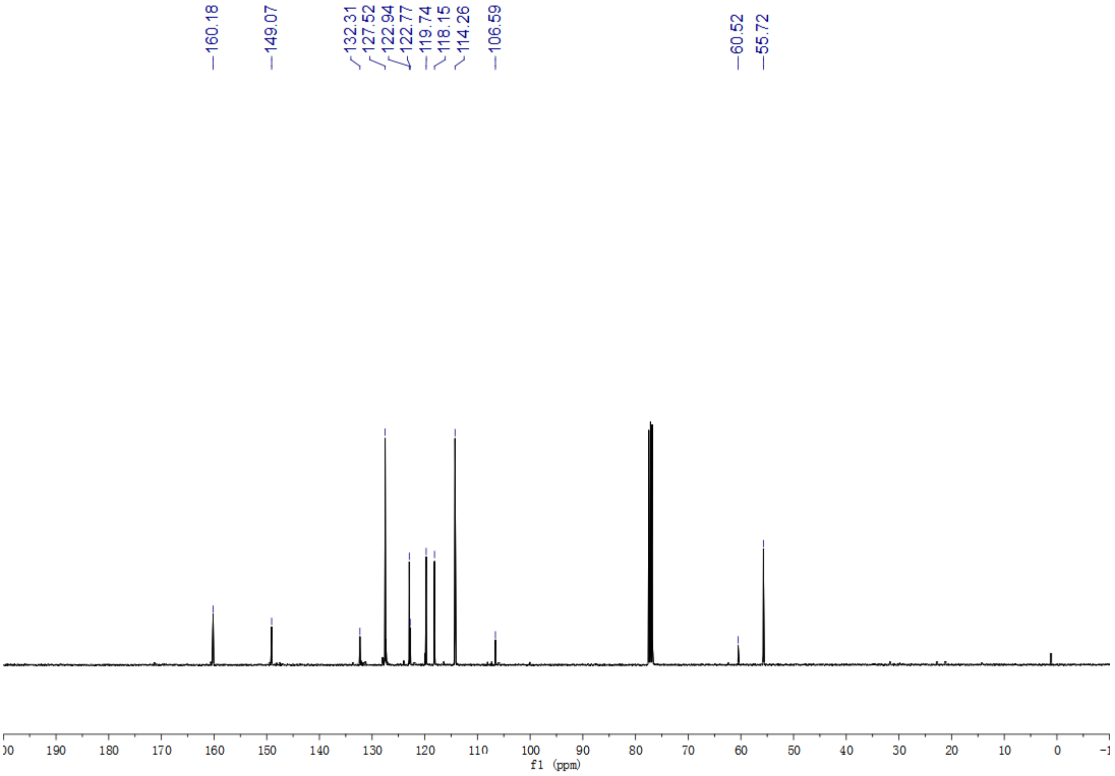

^{1}H NMR spectra for **2k**

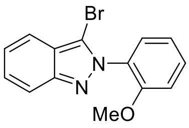

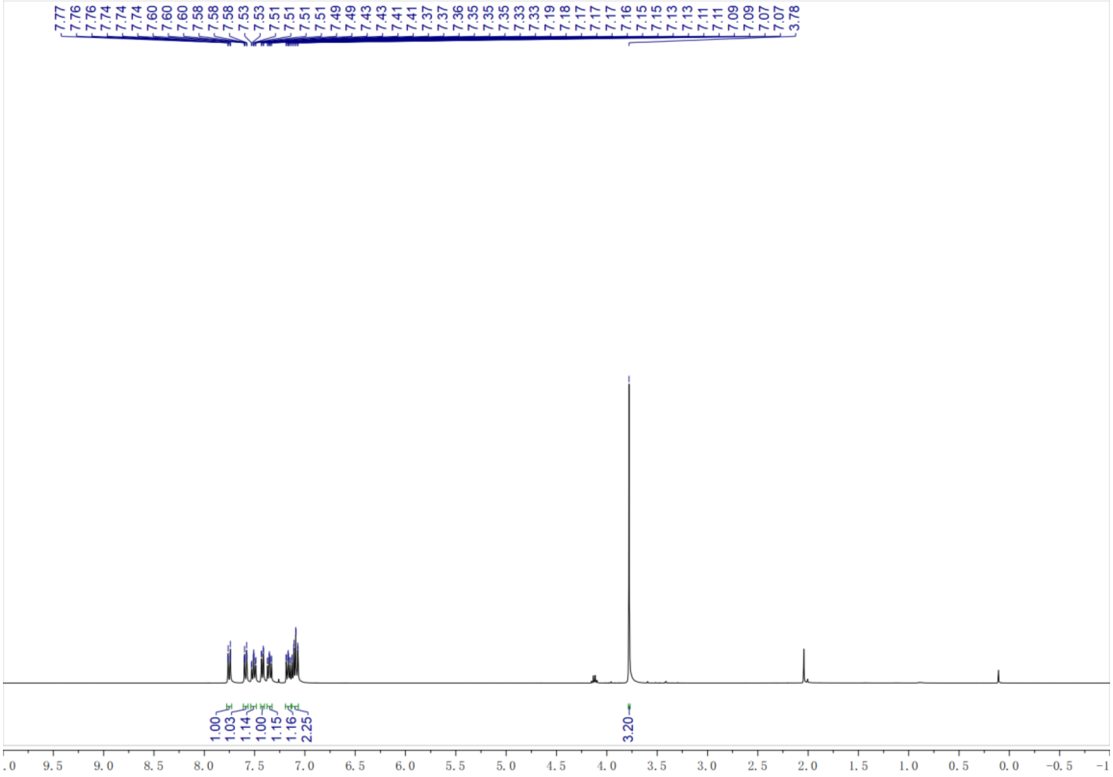

^{13}C NMR spectra for **2k**

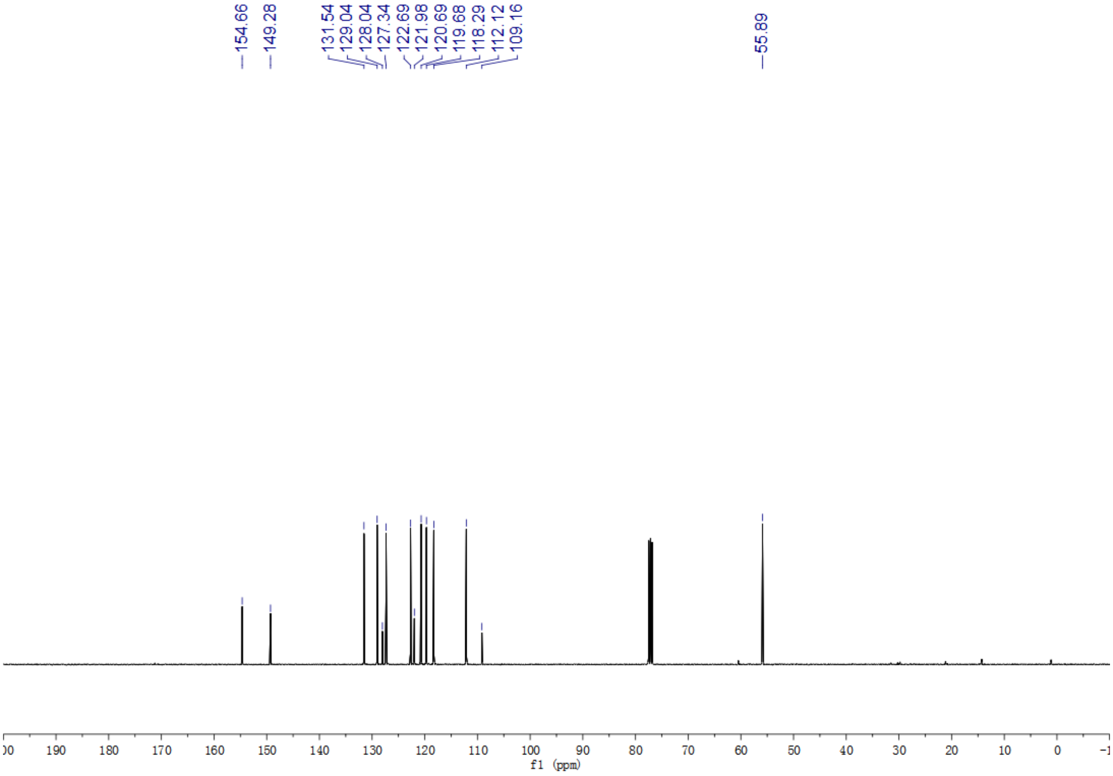

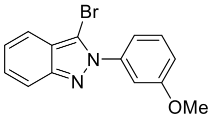

^{1}H NMR spectra for **2l**

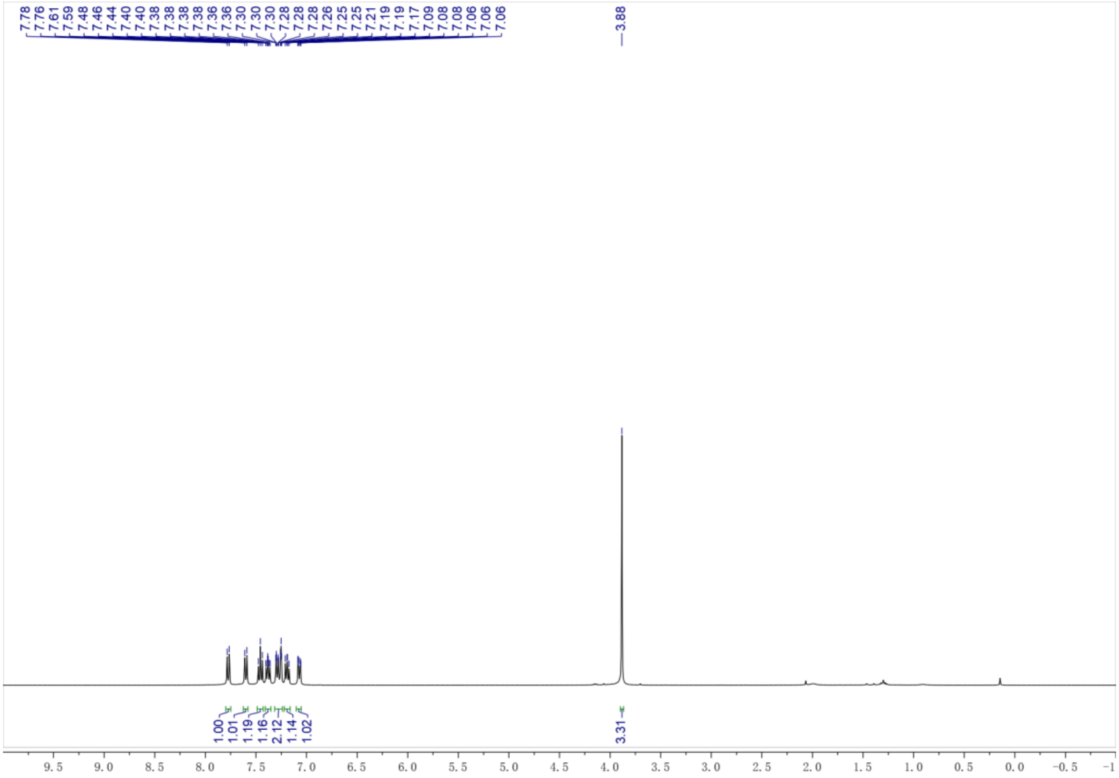

^{13}C NMR spectra for **2l**

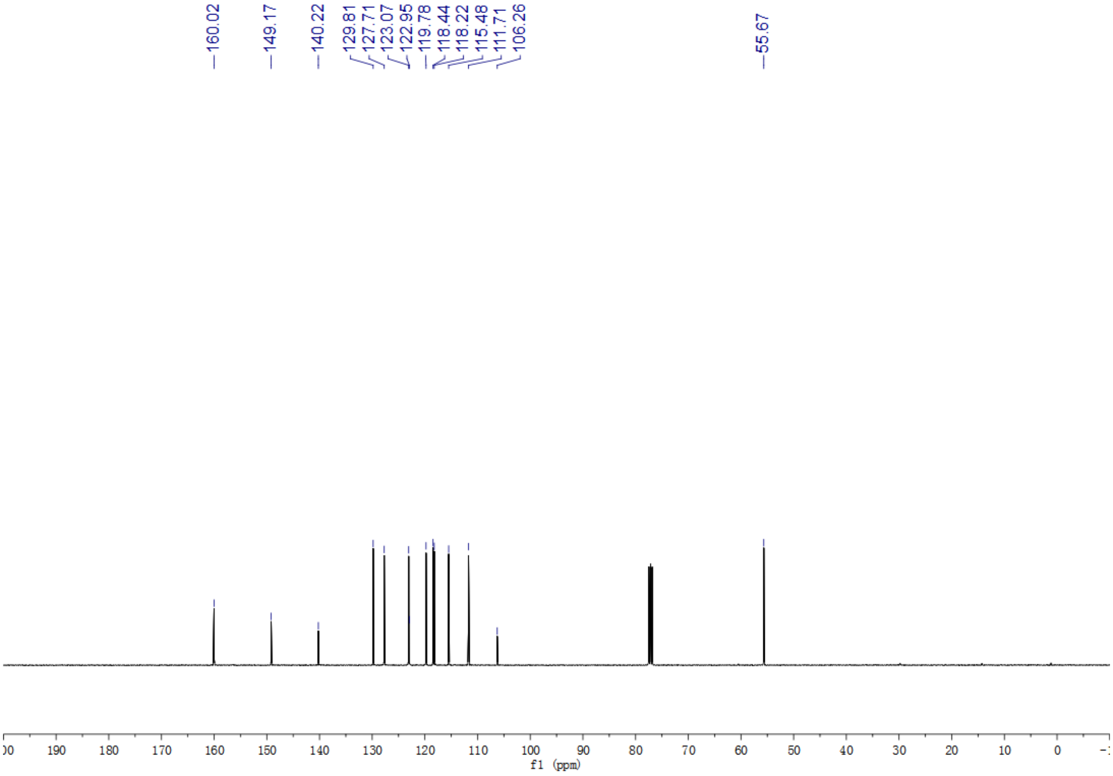

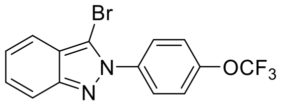

^{1}H NMR spectra for **2m**

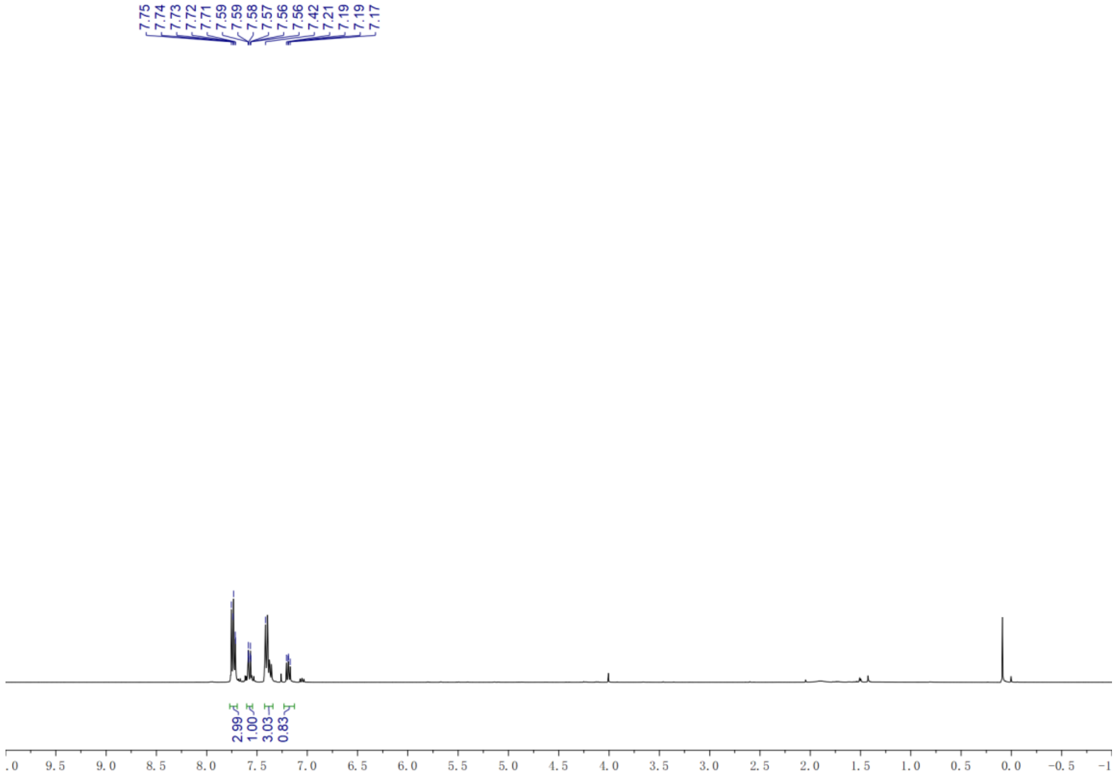

^{13}C NMR spectra for **2m**

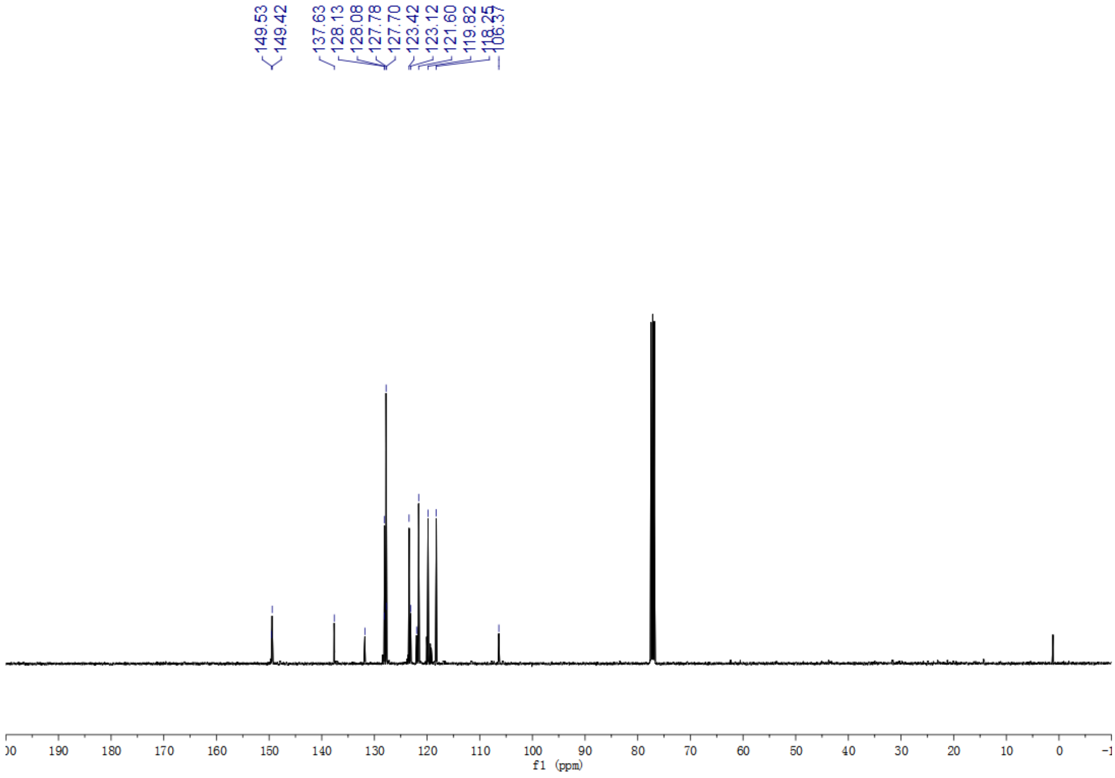

^{1}H NMR spectra for **2n**

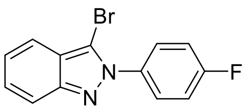

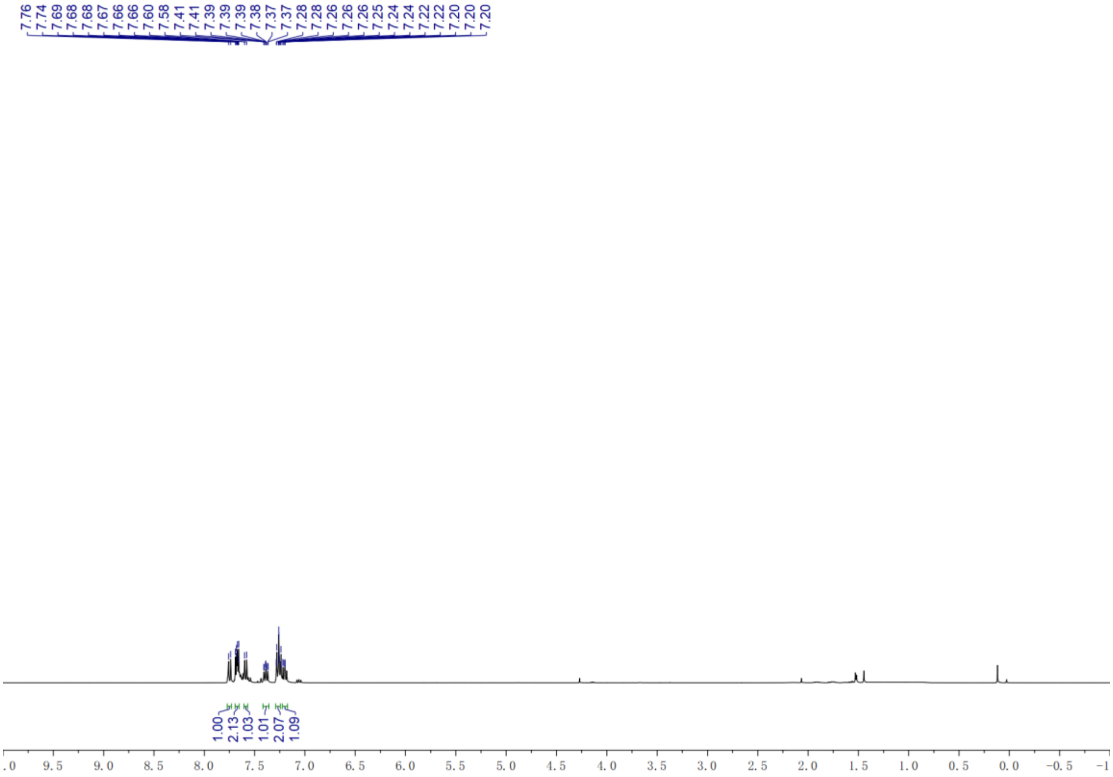

^{13}C NMR spectra for **2n**

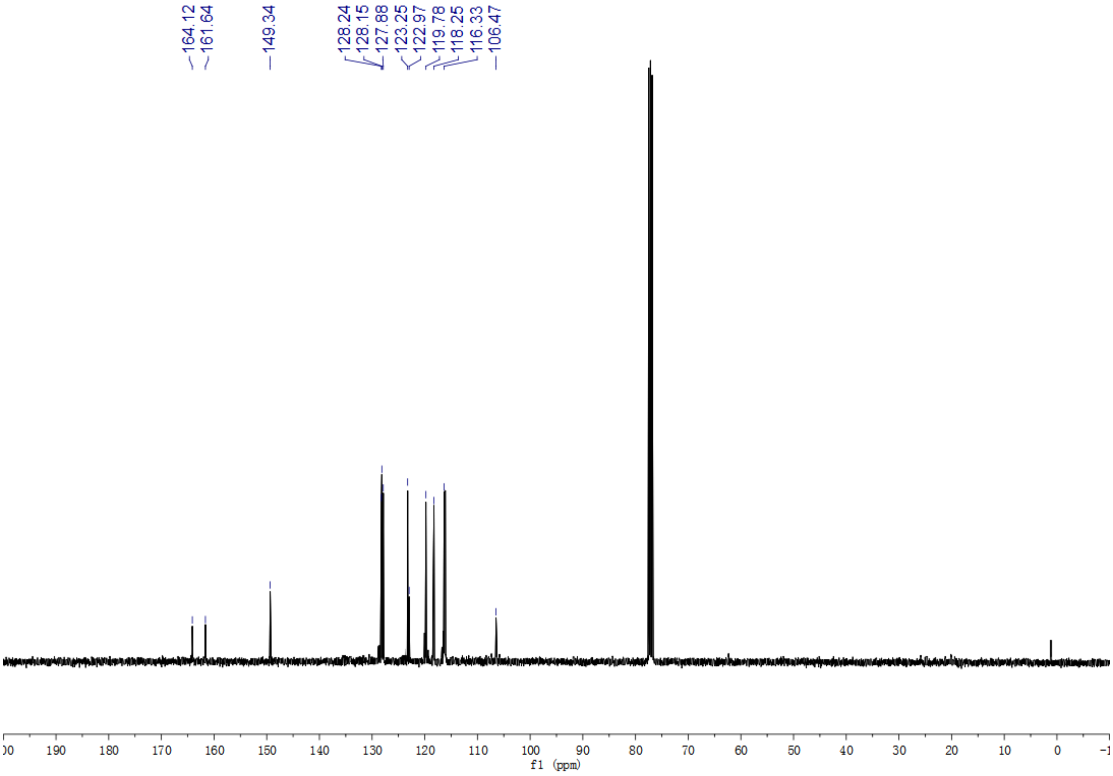

^{1}H NMR spectra for **2o**

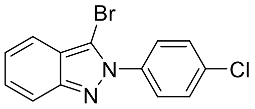

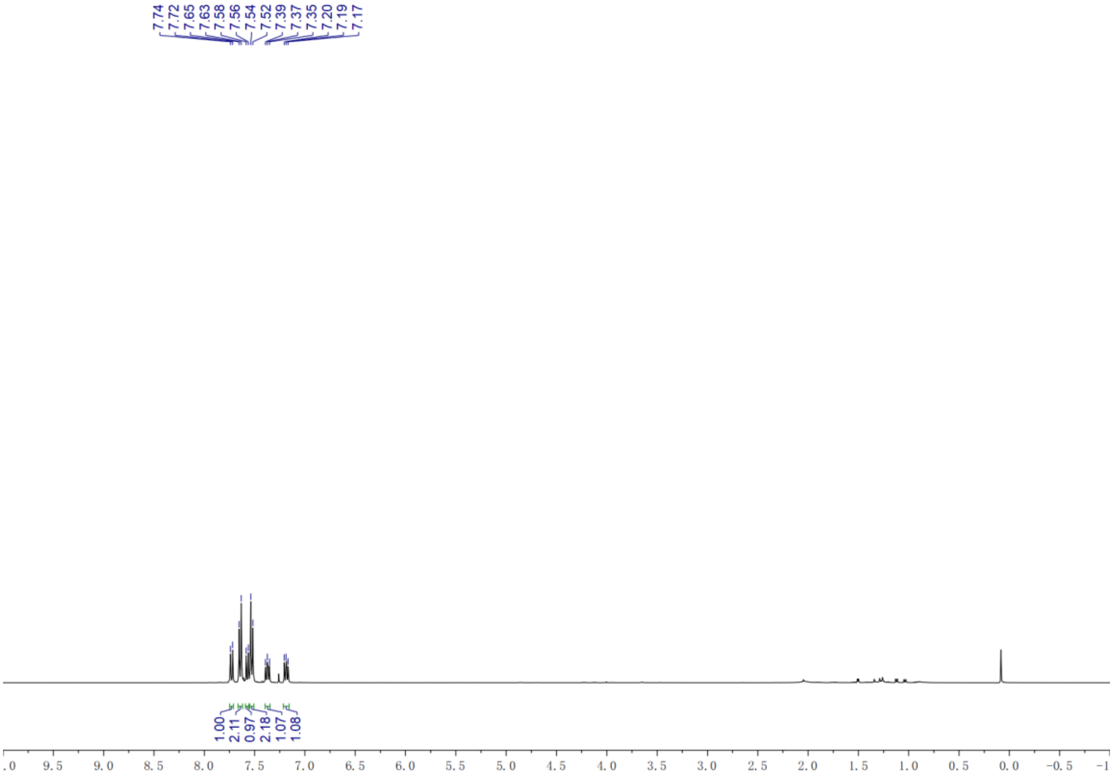

^{13}C NMR spectra for **2o**

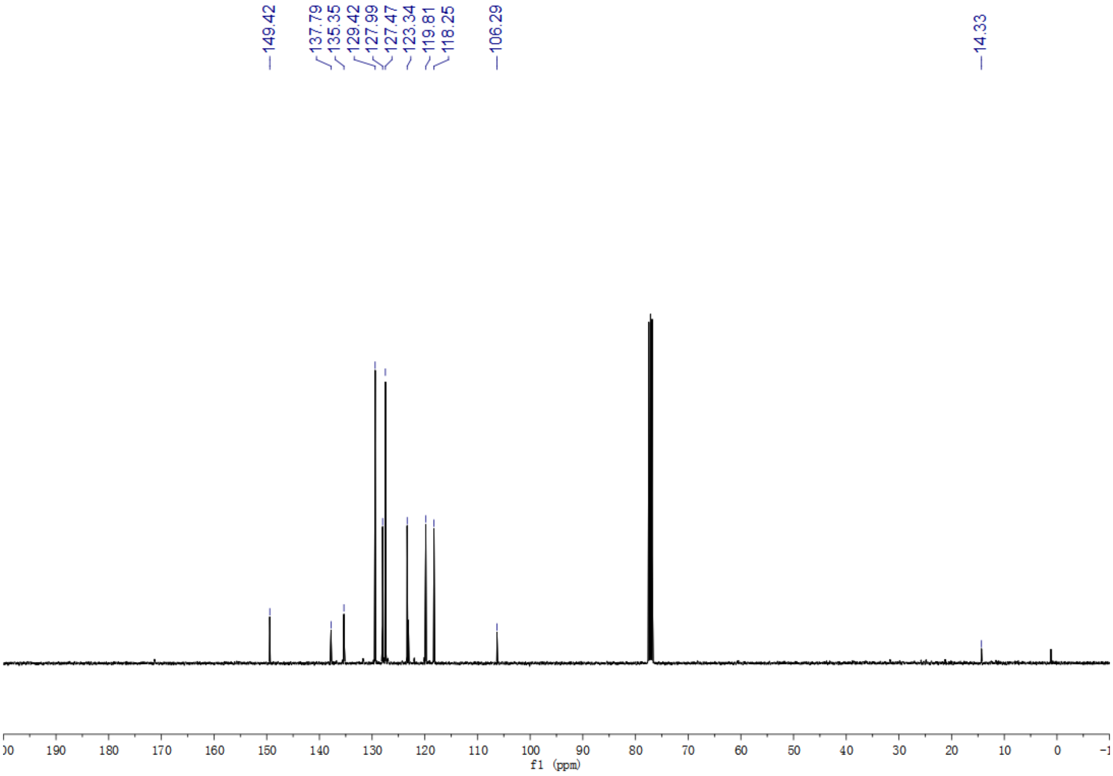

^{1}H NMR spectra for **2p**

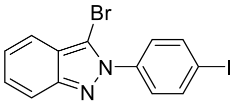

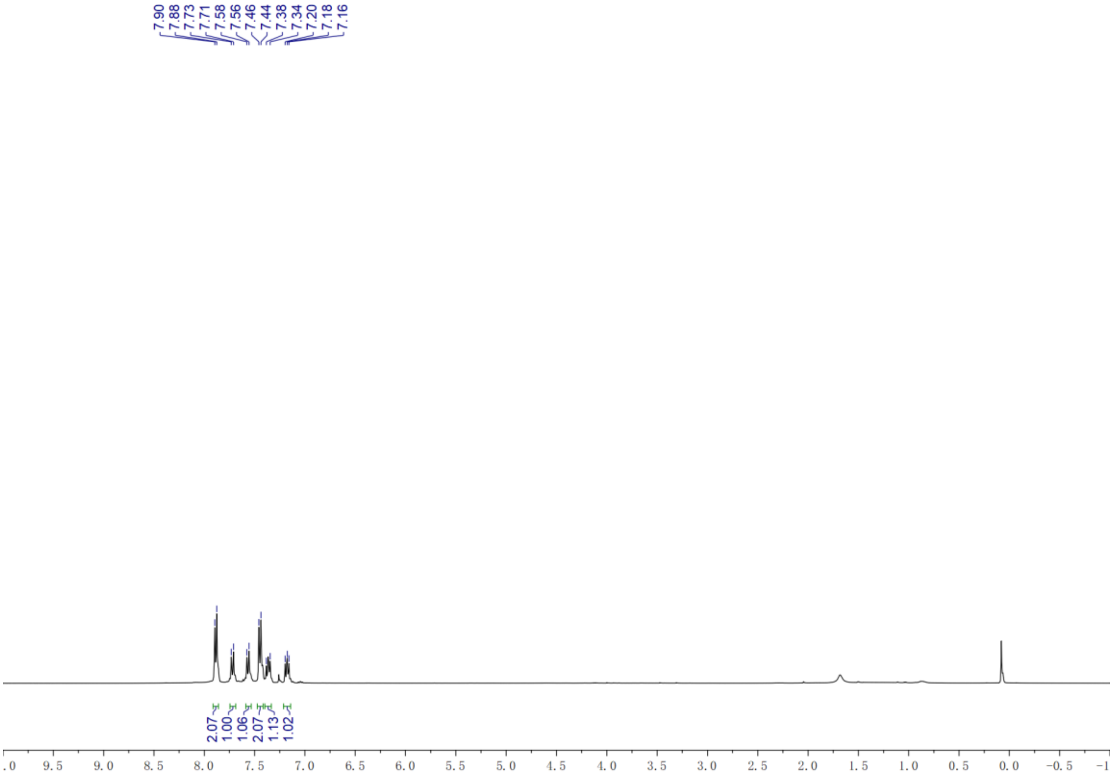

^{13}C NMR spectra for **2p**

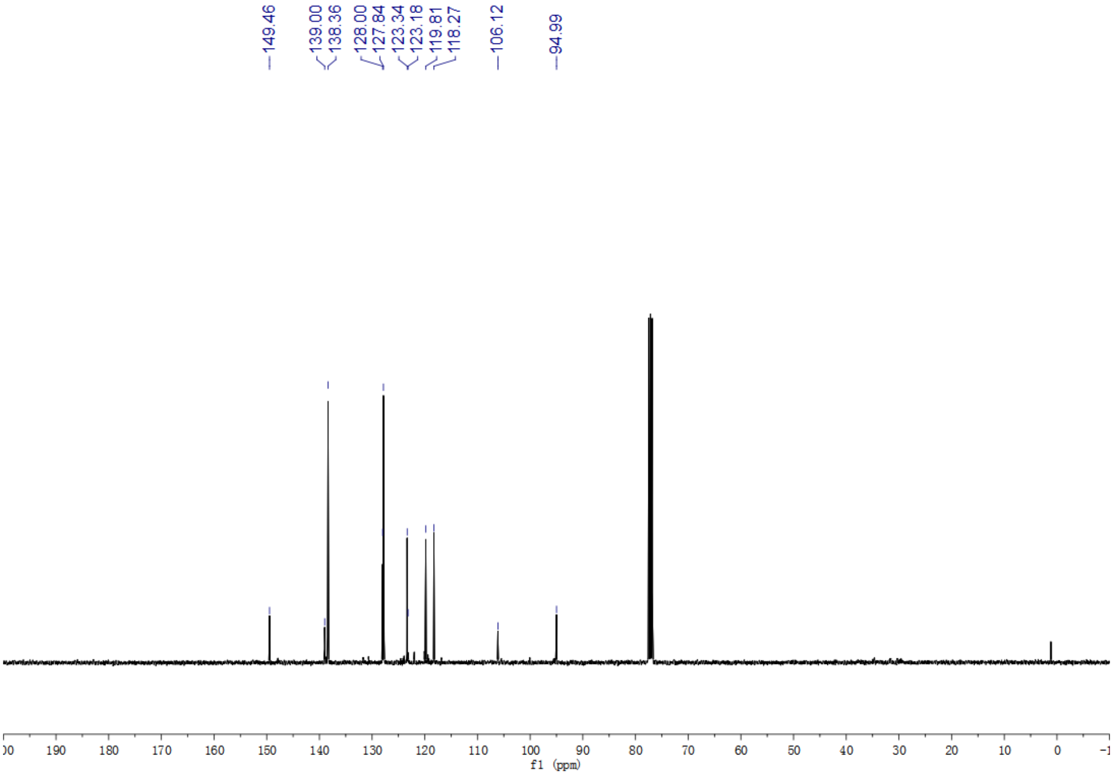

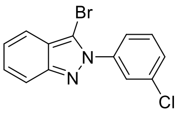

^{1}H NMR spectra for **2q**

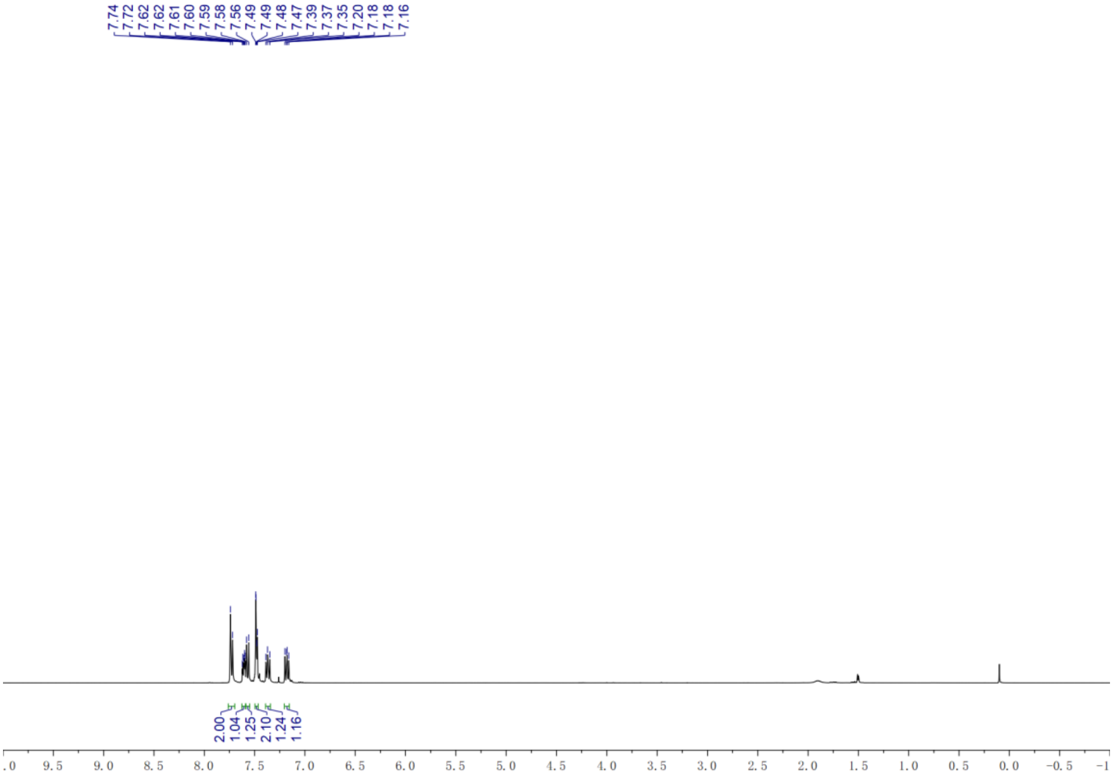

^{13}C NMR spectra for **2q**

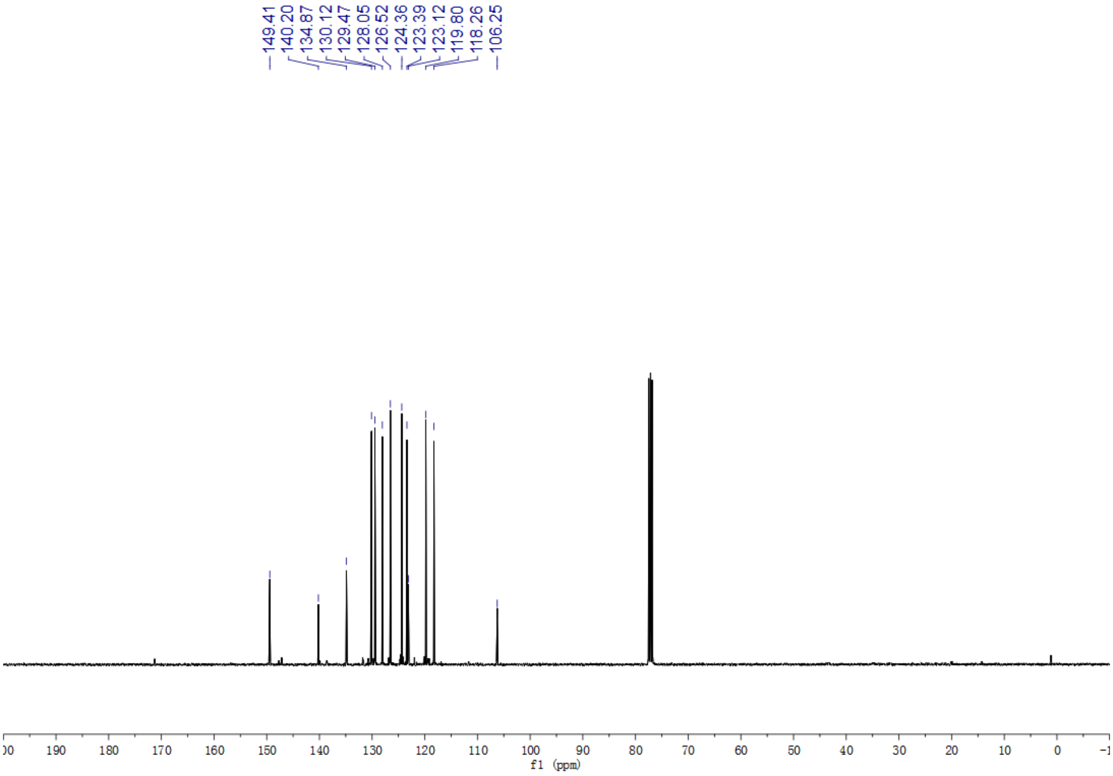

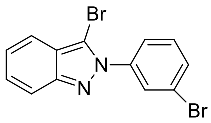

^{1}H NMR spectra for **2r**

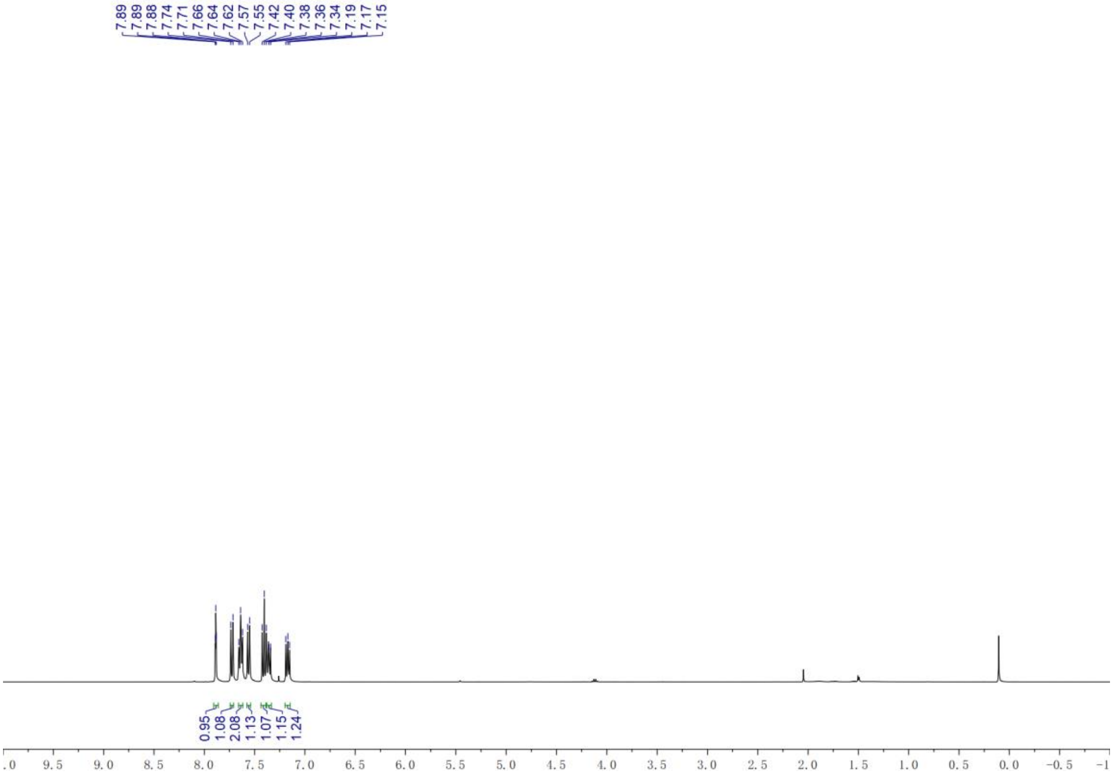

^{13}C NMR spectra for **2r**

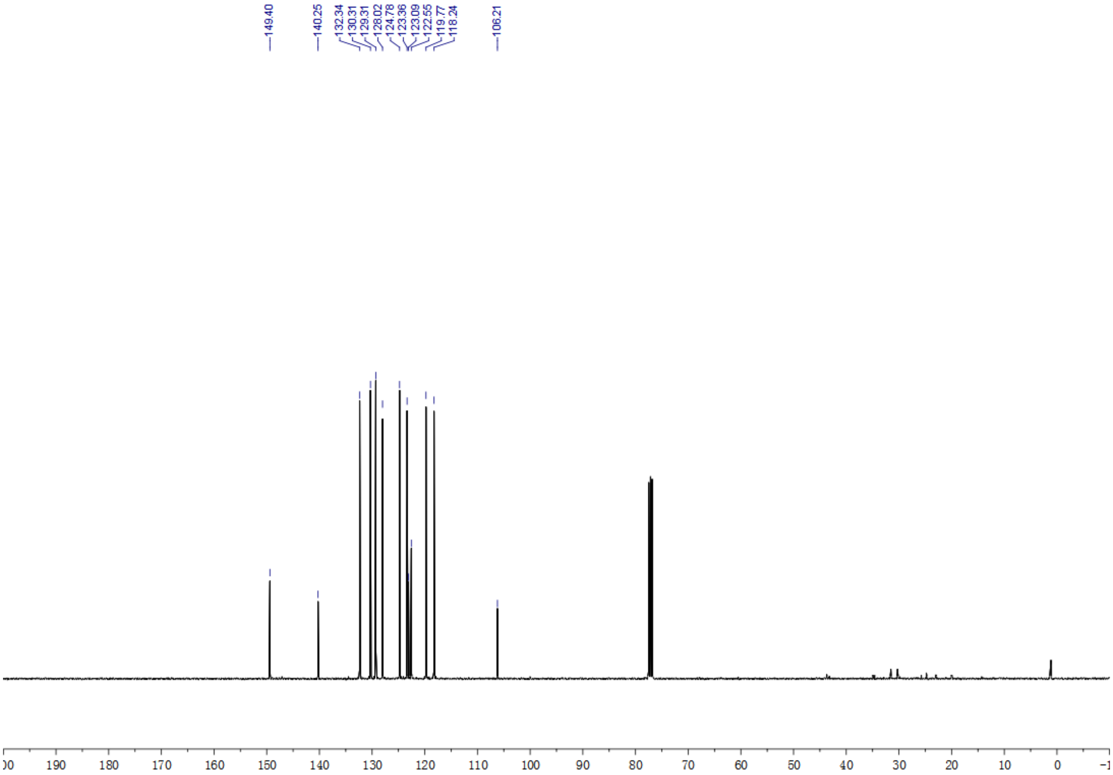

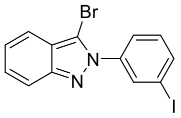

^{1}H NMR spectra for **2s**

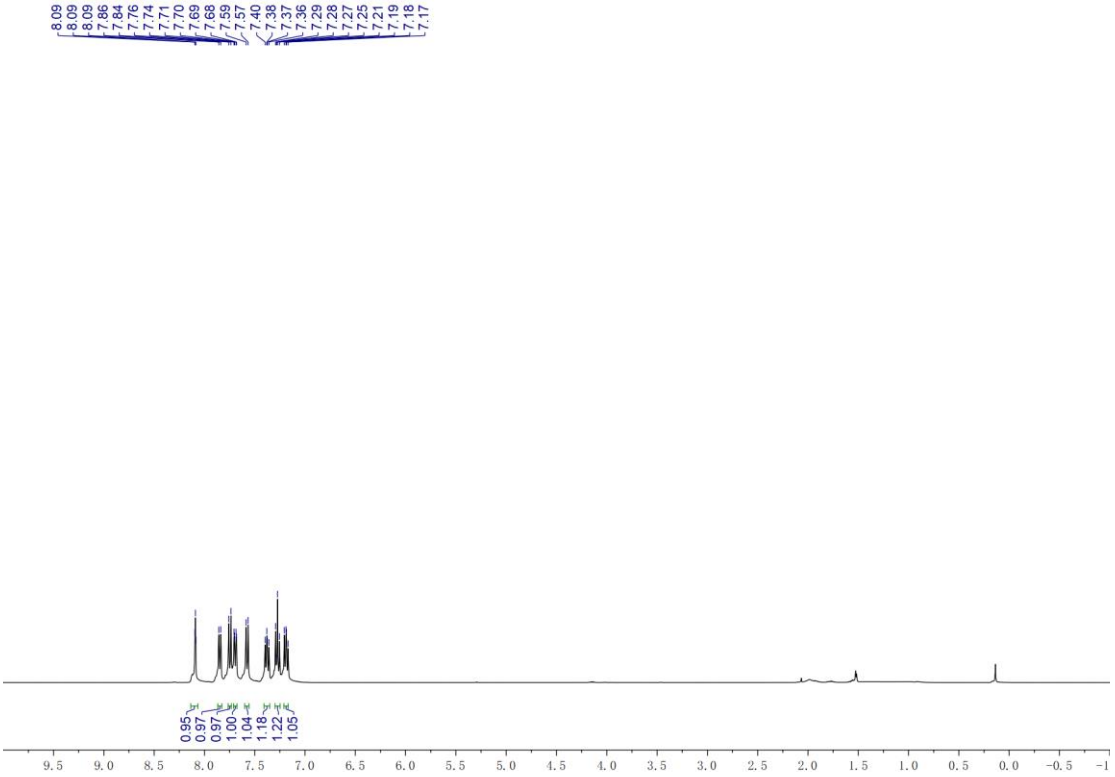

^{13}C NMR spectra for **2s**

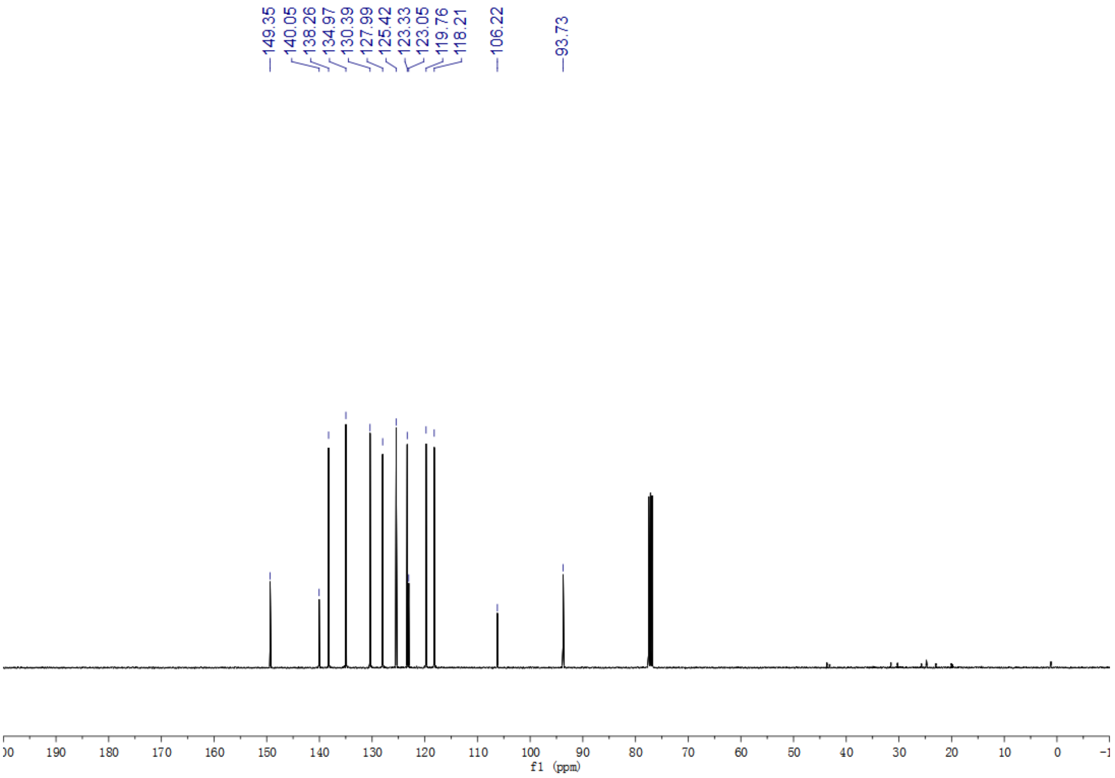

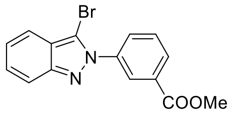

^{1}H NMR spectra for **2t**

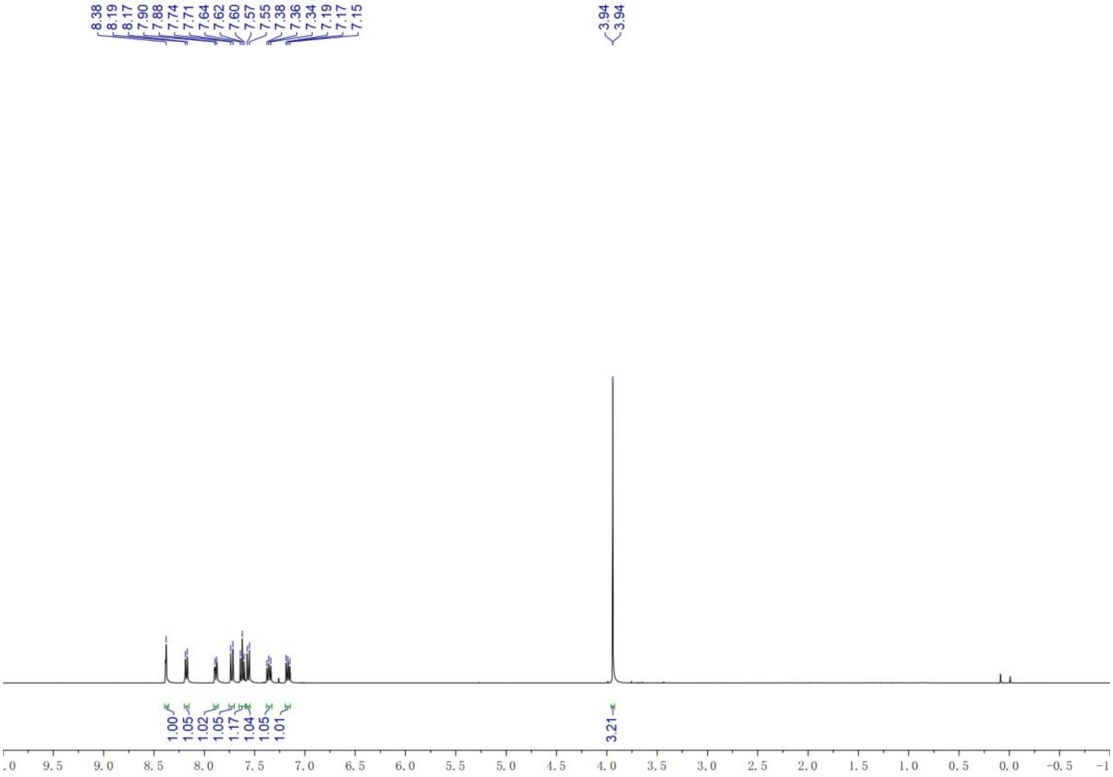

^{13}C NMR spectra for **2t**

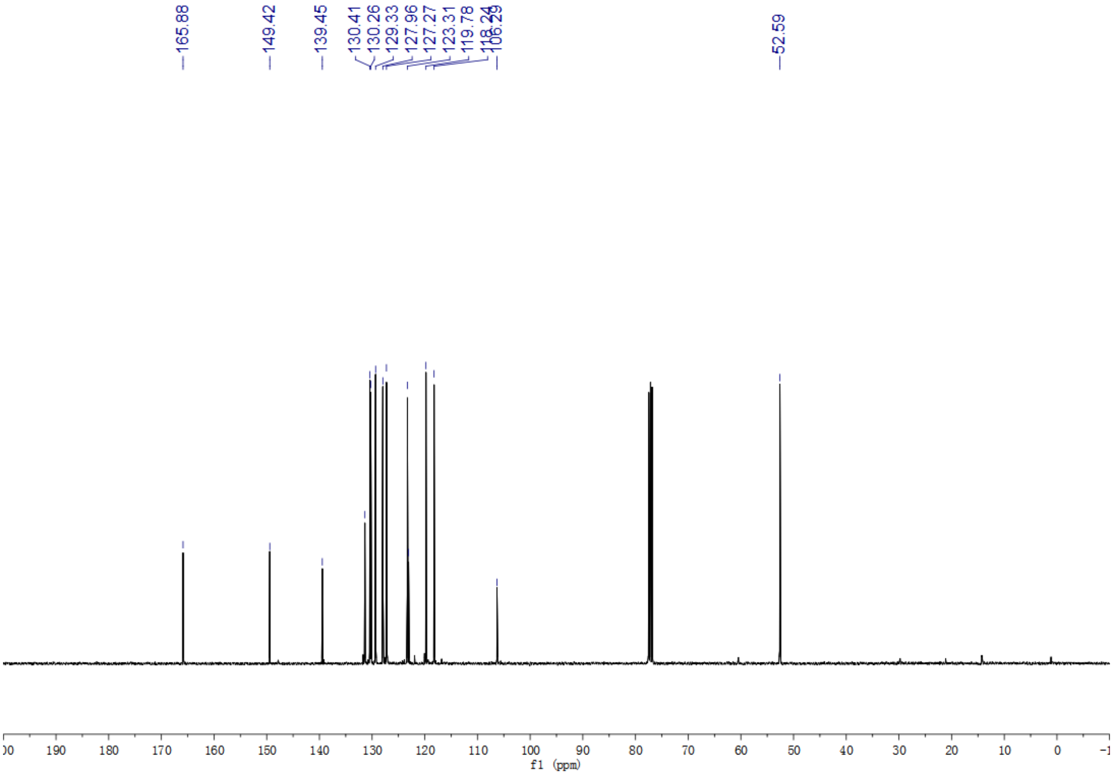

^{1}H NMR spectra for **2u**

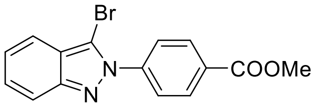

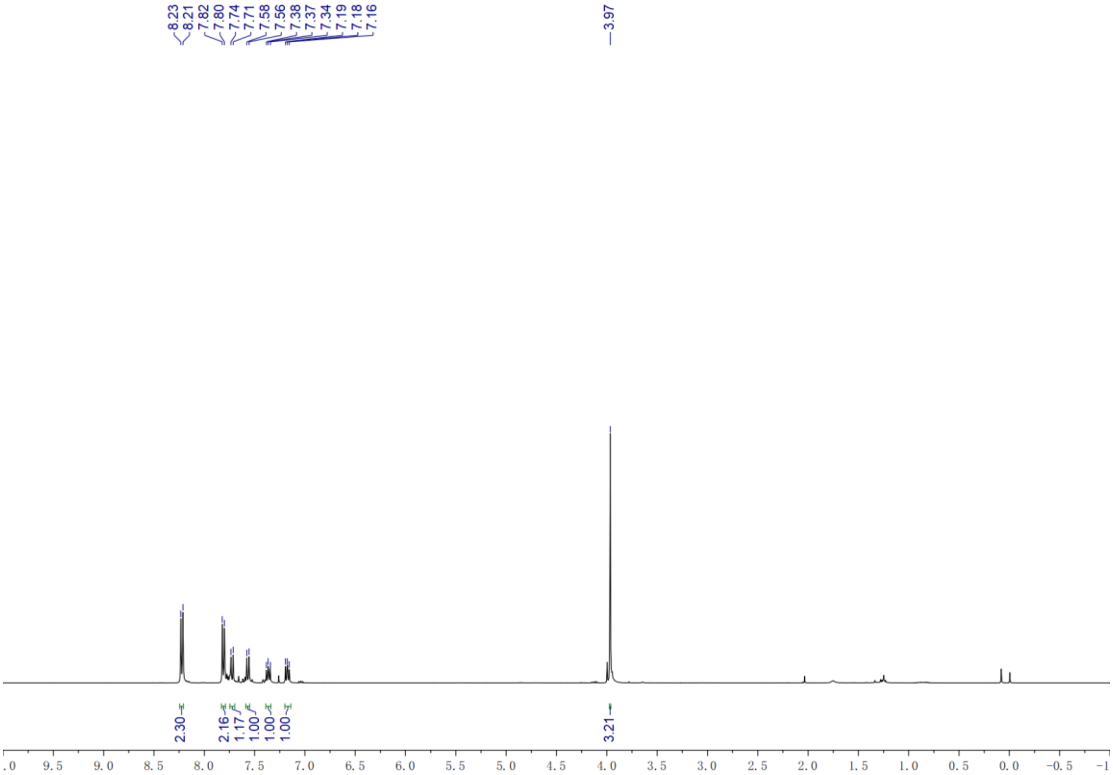

^{13}C NMR spectra for **2u**

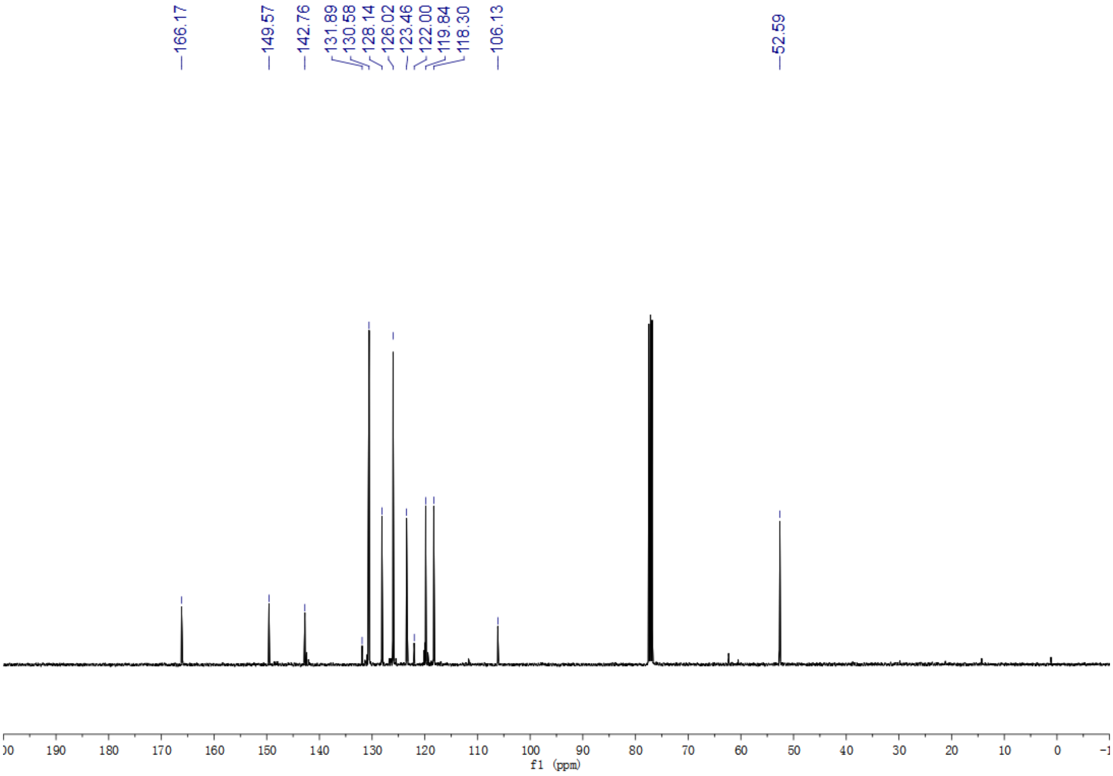

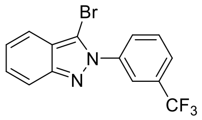

^{1}H NMR spectra for **2v**

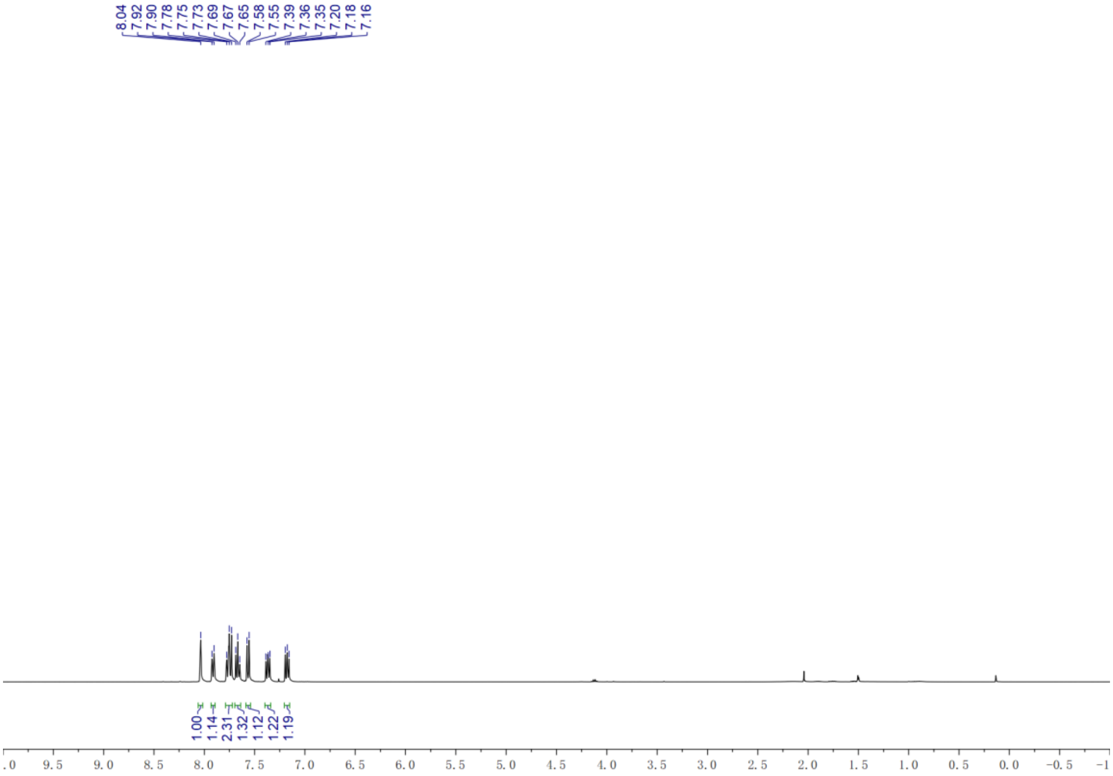

^{13}C NMR spectra for **2v**

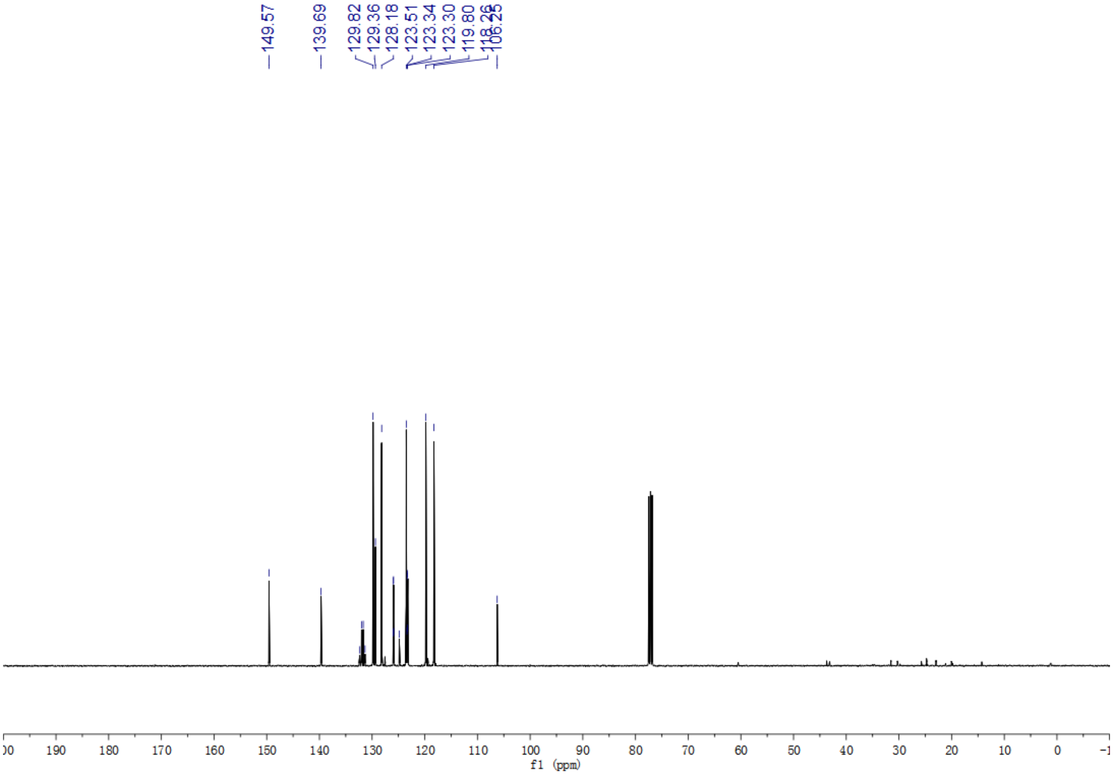

^{1}H NMR spectra for **2w**

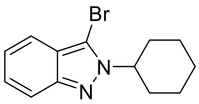

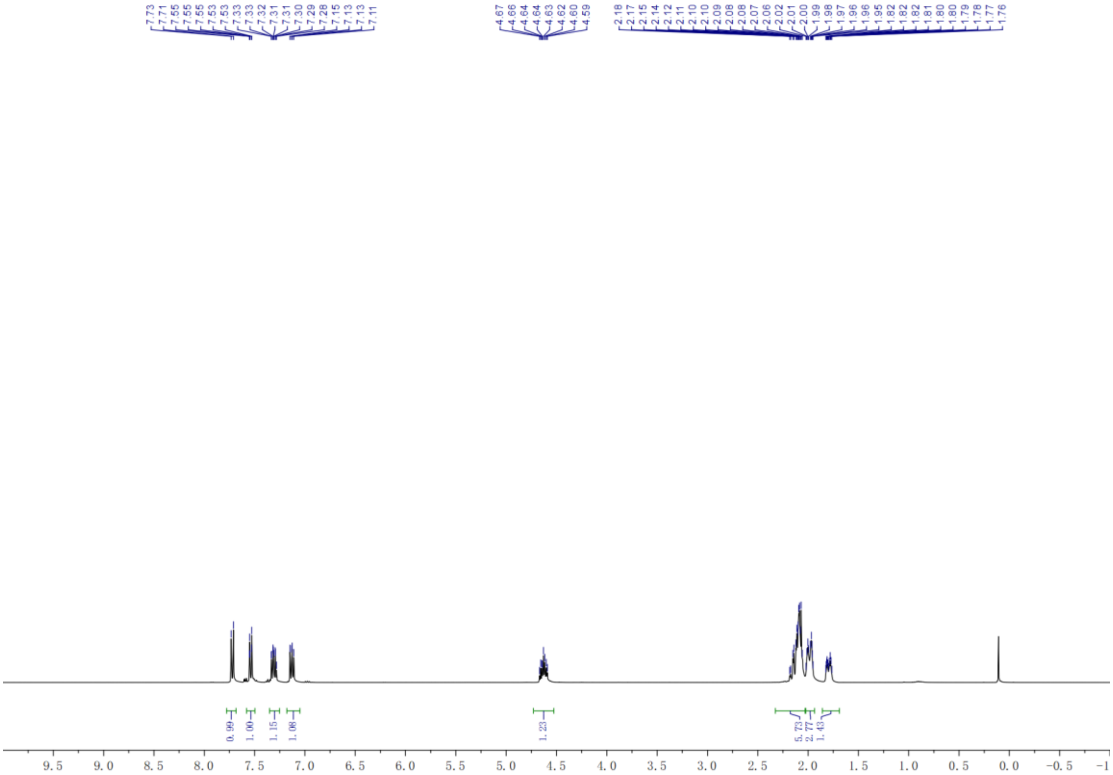

^{13}C NMR spectra for **2w**

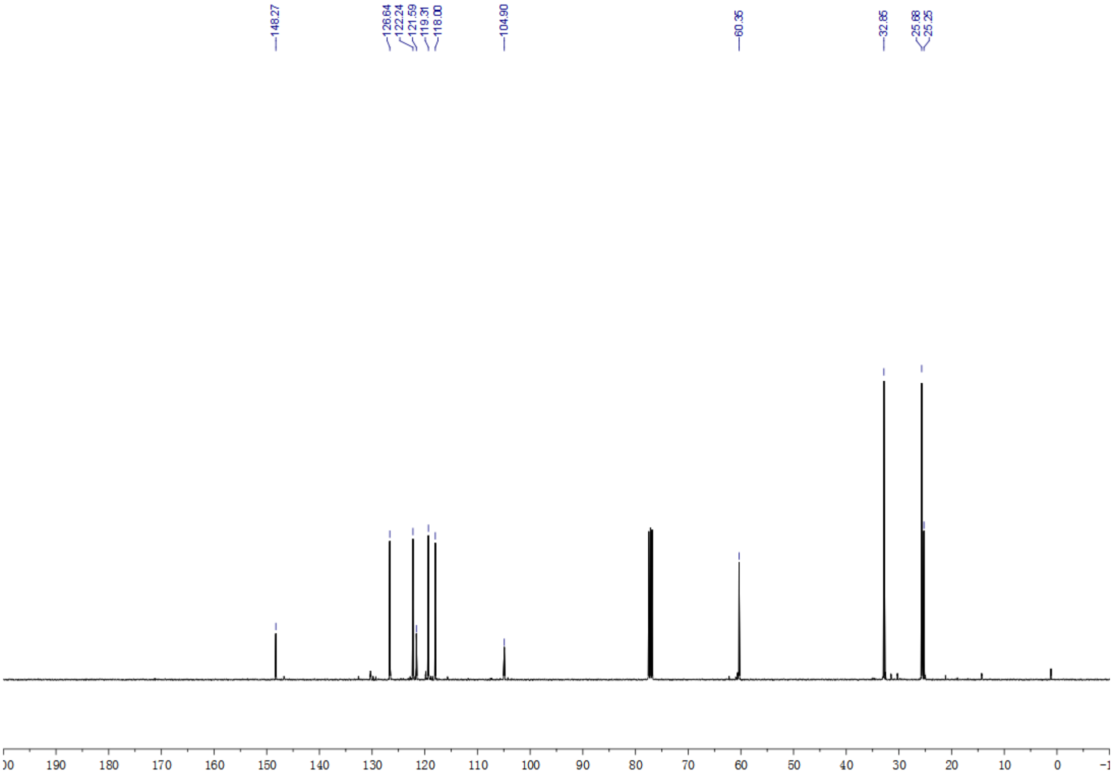

^{1}H NMR spectra for **2x**

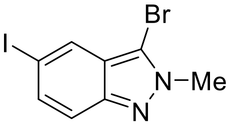

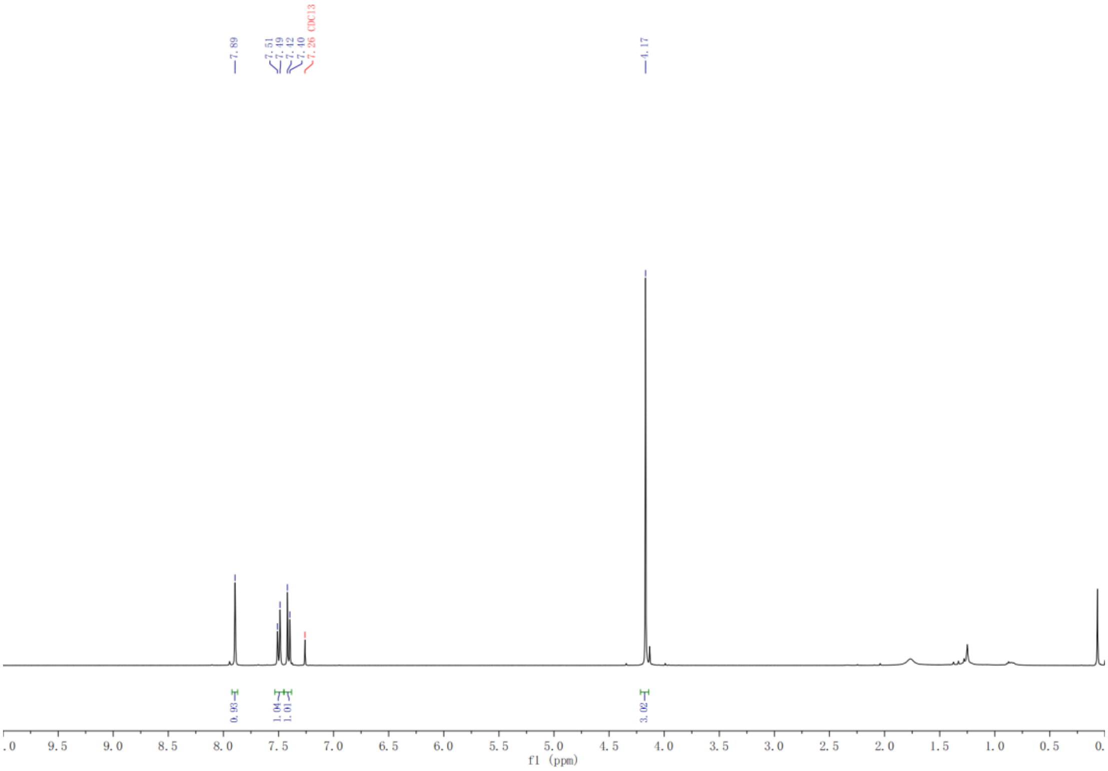

^{13}C NMR spectra for **2x**

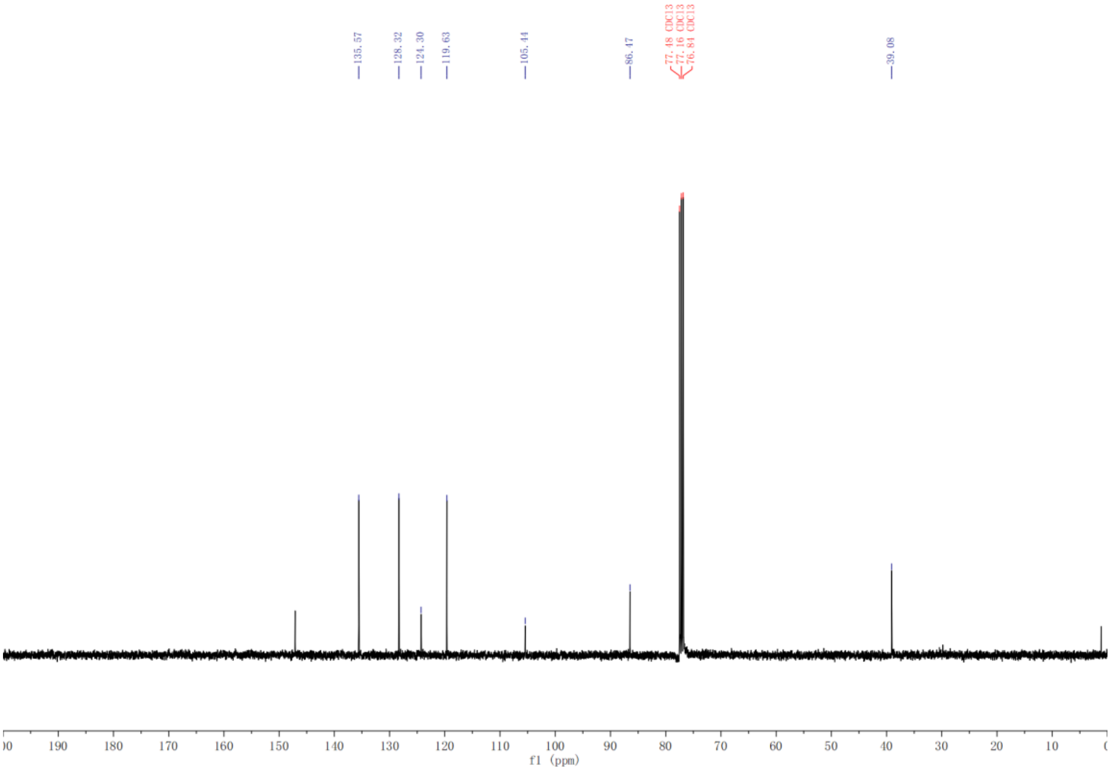

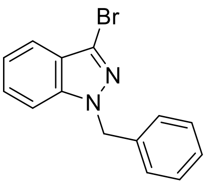

^{1}H NMR spectra for **2y**

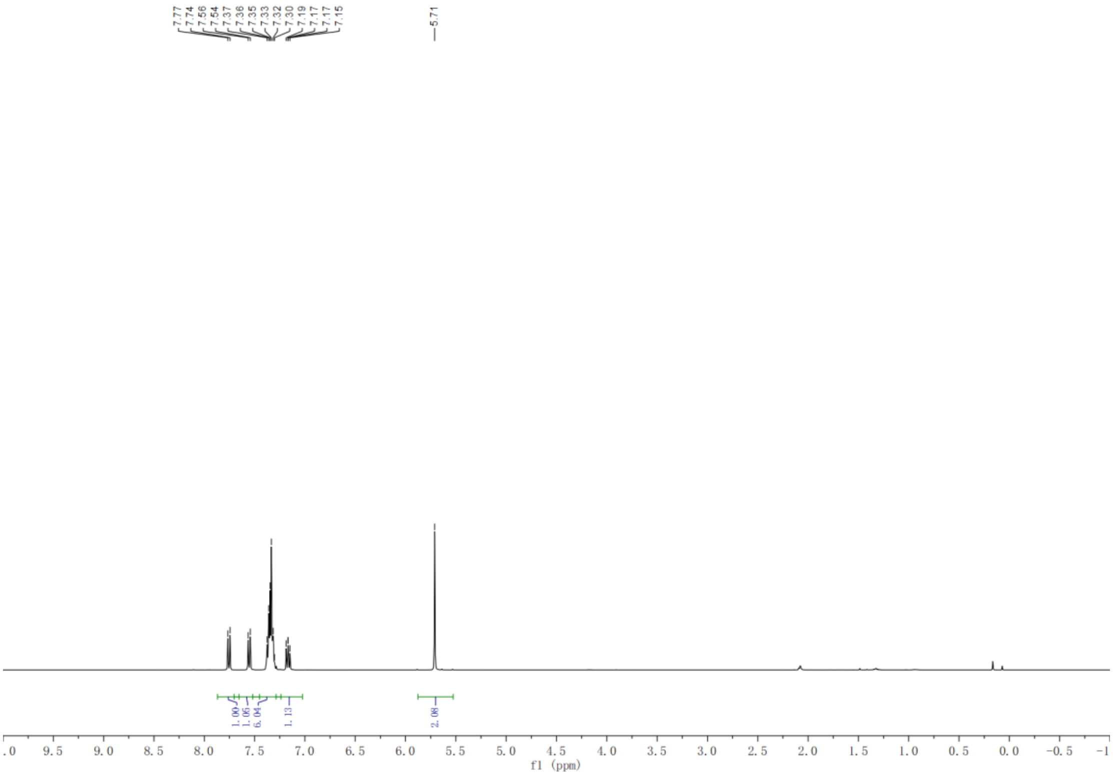

^{13}C NMR spectra for **2y**

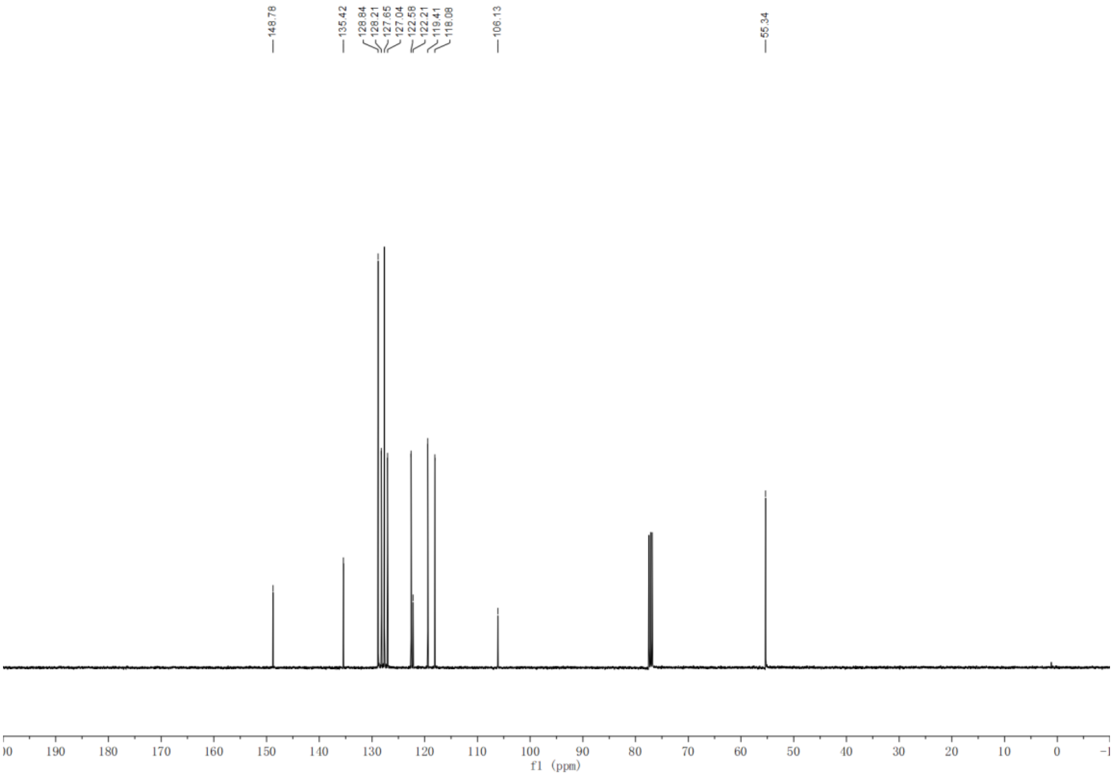

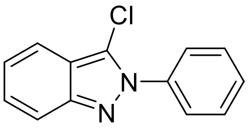

^{1}H NMR spectra for **2z**

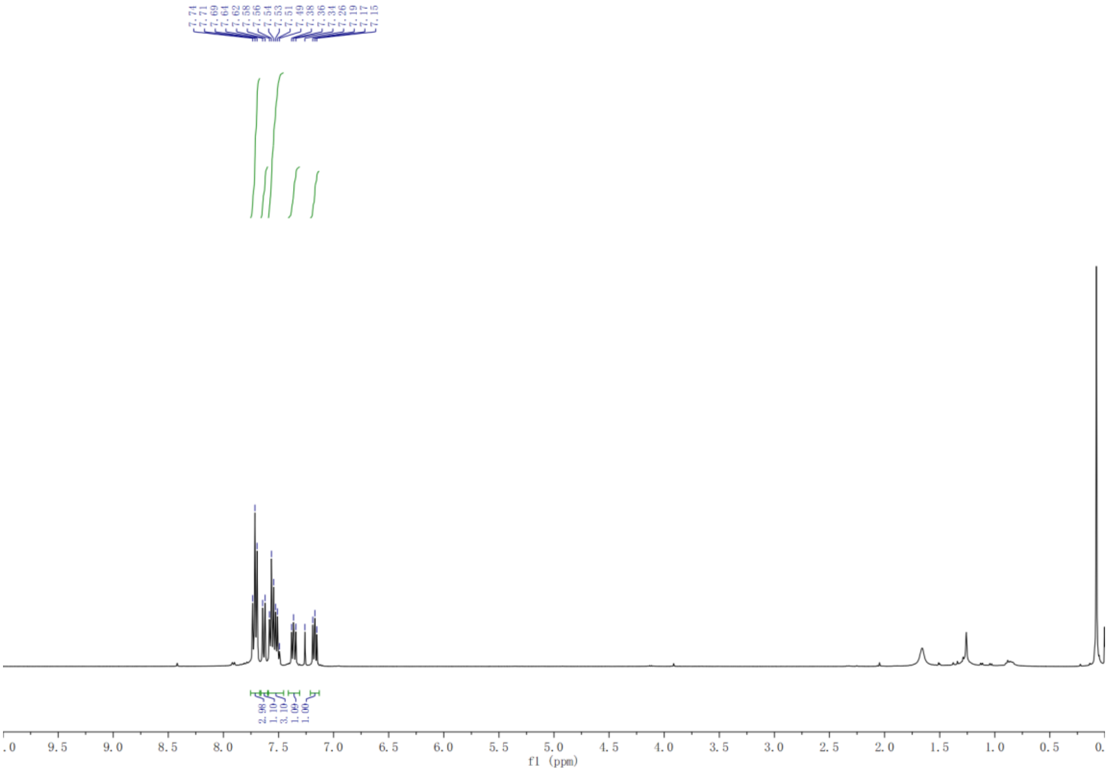

^{13}C NMR spectra for **2z**

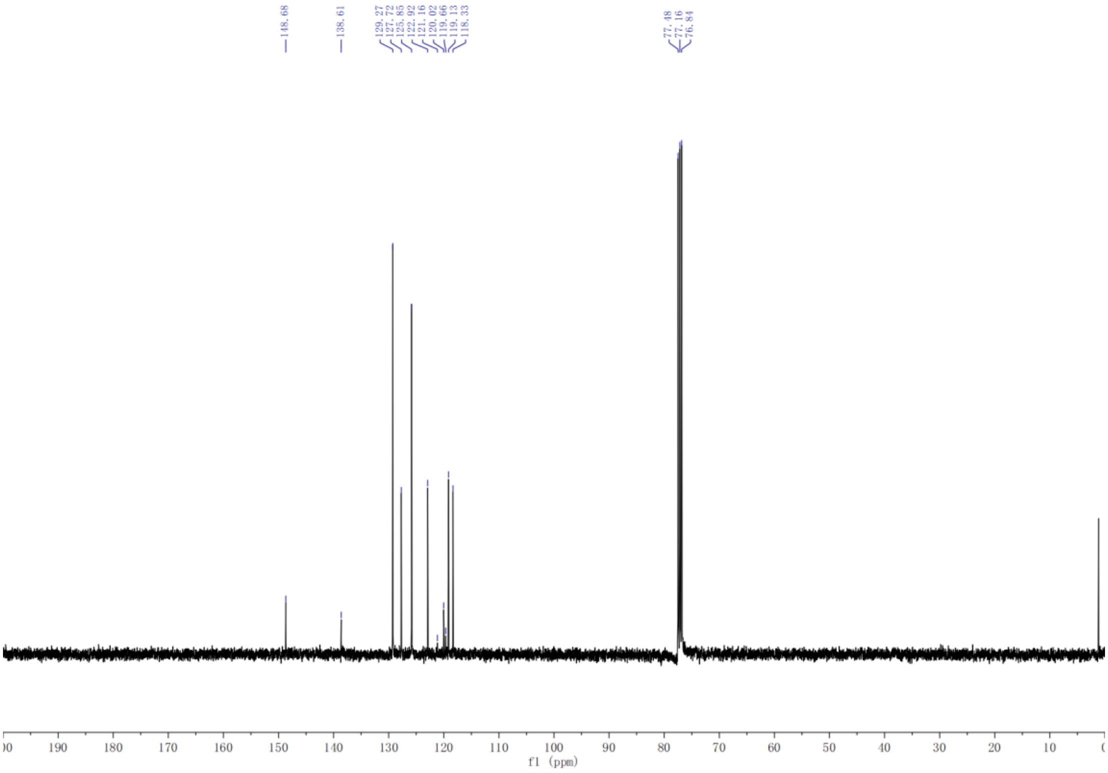

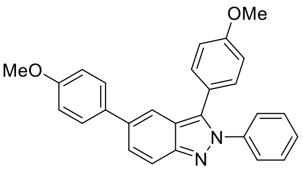

^{1}H NMR spectra for **4**

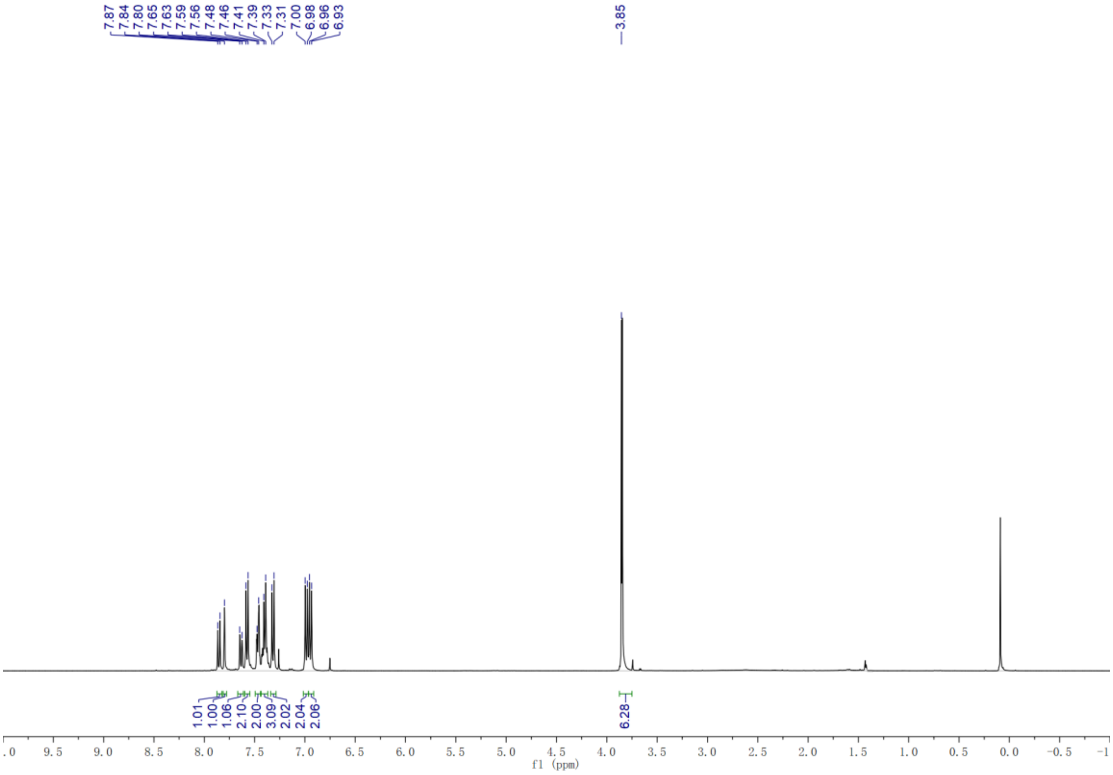

^{13}C NMR spectra for **4**

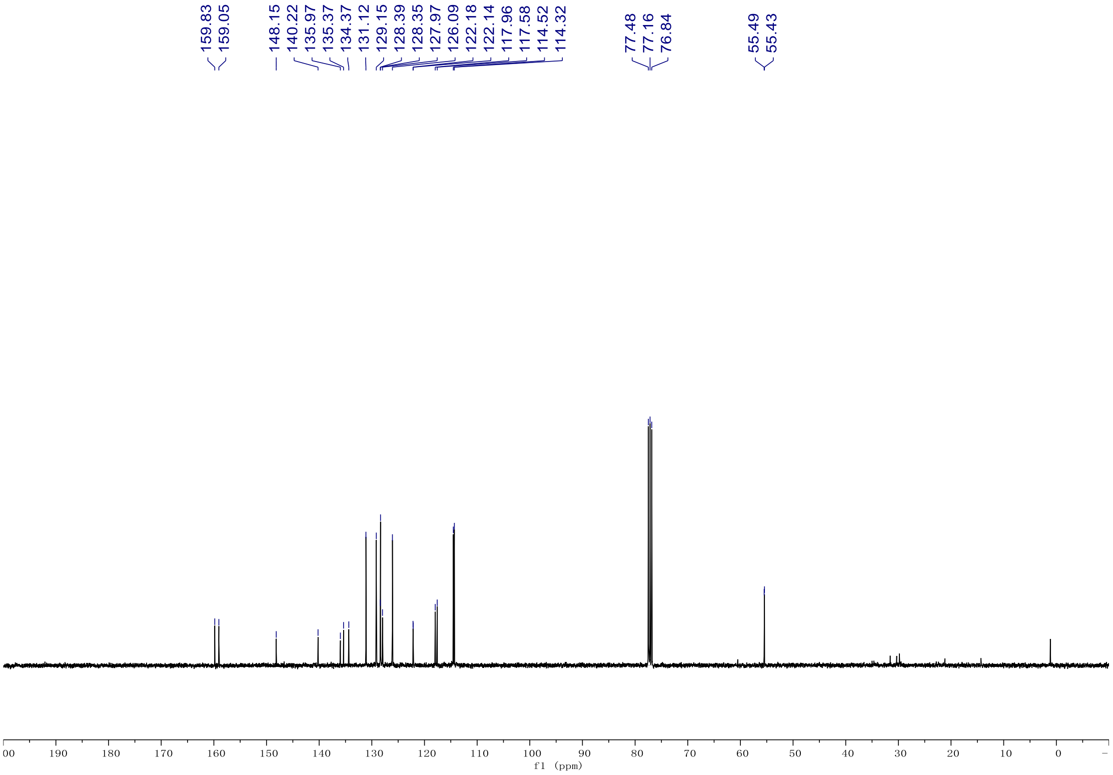

^{1}H NMR spectra for **3a** 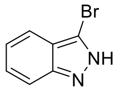

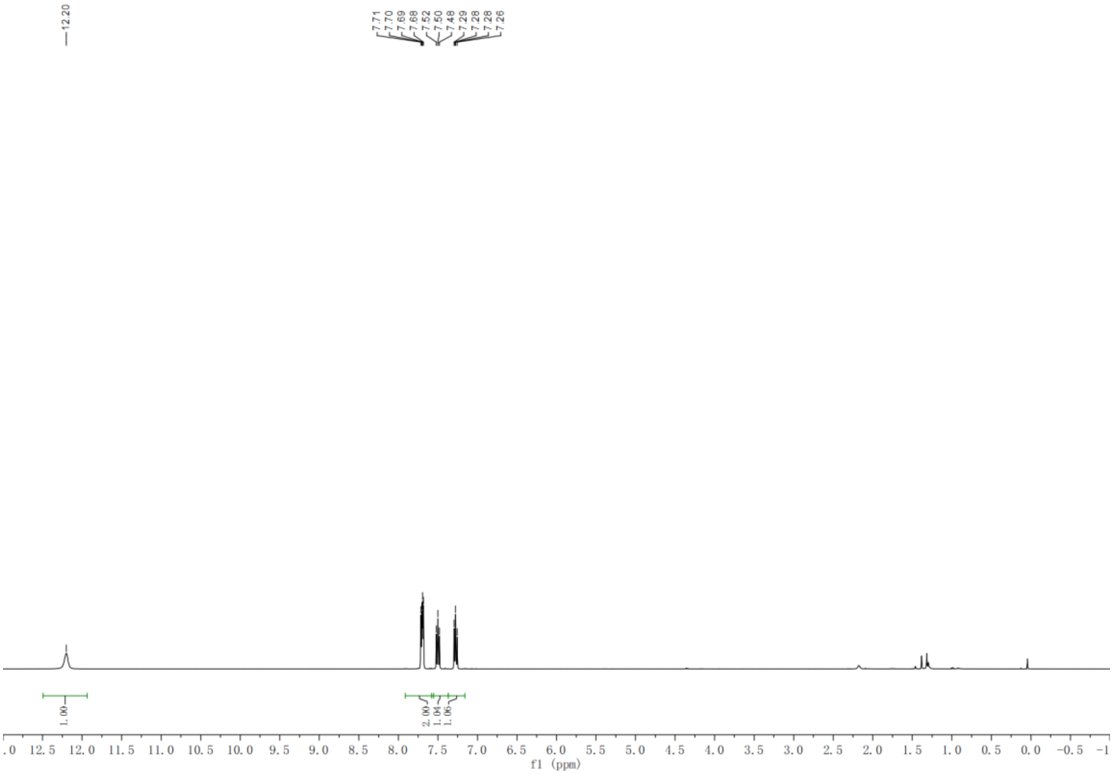

^{13}C NMR spectra for **3a**

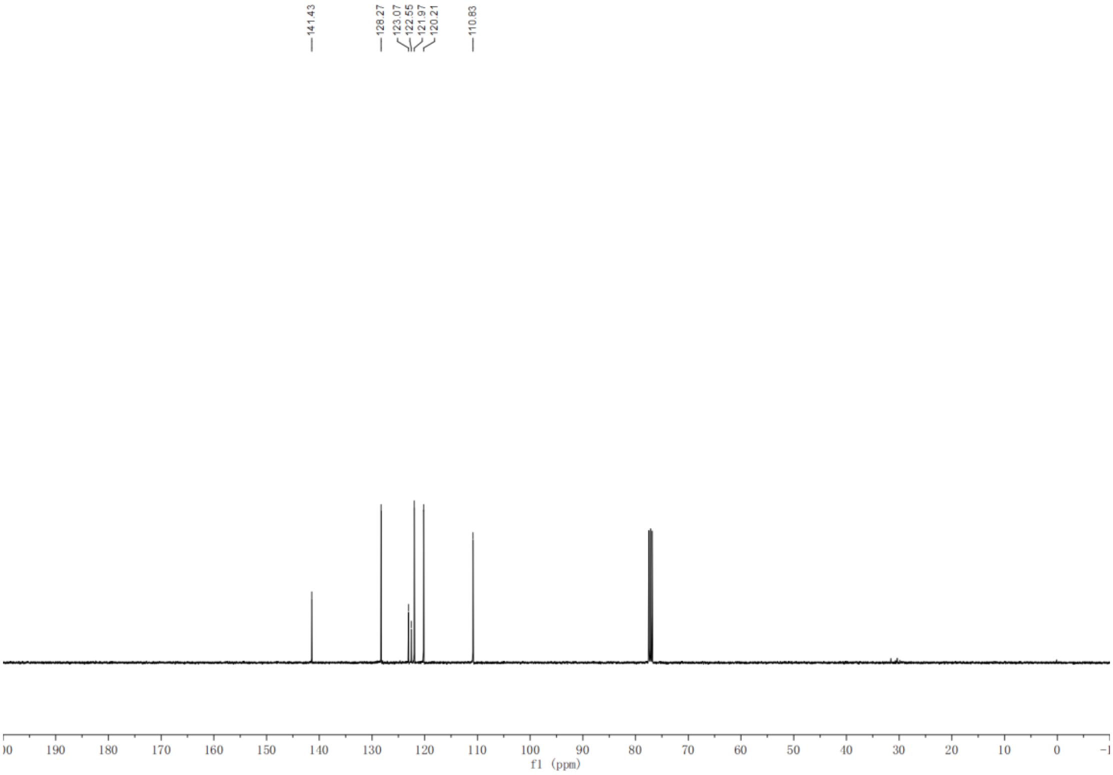

Supplement: RA-013-D2RA06867B-s001 [file RA-013-D2RA06867B-s001.pdf]
